# Initiation and completion rates for latent tuberculosis infection treatment: a systematic review

**DOI:** 10.1186/s12879-016-1550-y

**Published:** 2016-05-17

**Authors:** Andreas Sandgren, Marije Vonk Noordegraaf-Schouten, Femke van Kessel, Anke Stuurman, Anouk Oordt-Speets, Marieke J. van der Werf

**Affiliations:** Former Surveillance and Response Section, European Centre for Disease Prevention and Control (ECDC), Stockholm, 171 65 Sweden; Pallas, Health Research and Consultancy B.V., Rotterdam, 3001 The Netherlands; Tomtebodavägen 11a, 171 65 Solna, Sweden

**Keywords:** Tuberculosis, Latent tuberculosis, Treatment initiation, Treatment completion, Risk groups

## Abstract

**Background:**

Control of latent tuberculosis infection (LTBI) is an important step towards tuberculosis elimination. Preventive treatment will prevent the development of disease in most cases diagnosed with LTBI. However, low initiation and completion rates affect the effectiveness of preventive treatment. The objective was to systematically review data on initiation rates and completion rates for LTBI treatment regimens in the general population and specific populations with LTBI.

**Methods:**

A systematic review of the literature (PubMed, Embase) published up to February 2014 was performed.

**Results:**

Forty-five studies on initiation rates and 83 studies on completion rates of LTBI treatment were found. These studies provided initiation rates (IR) and completion rates (CR) in people with LTBI among the general population (IR 26–99 %, CR 39–96 %), case contacts (IR 40–95 %, CR 48–82 %), healthcare workers (IR 47–98 %, CR 17–79 %), the homeless (IR 34–90 %, CR 23–71 %), people who inject drugs (IR 52–91 %, CR 38–89 %), HIV-infected individuals (IR 67–92 %, CR 55–95 %), inmates (IR 7–90 %, CR 4–100 %), immigrants (IR 23–97 %, CR 7–86 %), and patients with comorbidities (IR 82–93 %, CR 75–92 %). Generally, completion rates were higher for short than for long LTBI treatment regimens.

**Conclusion:**

Initiation and completion rates for LTBI treatment regimens were frequently suboptimal and varied greatly within and across different populations.

**Electronic supplementary material:**

The online version of this article (doi:10.1186/s12879-016-1550-y) contains supplementary material, which is available to authorized users.

## Background

In the European Union and European Economic Area (EU/EEA) 65 thousand cases of tuberculosis (TB) were reported in 2013, of which 77 % had pulmonary TB [1]. Cases with pulmonary TB produce microscopic droplets when coughing, sneezing, or spitting which can infect other individuals [2]. Exposure to *Mycobacterium tuberculosis* may result in latent TB infection (LTBI), a state in which the host immune system controls the replication of the bacillus to the extent that the progression to TB disease is prevented [3, 4]. In a later phase, LTBI may progress to TB disease, especially if the immune system is compromised [3, 5]. Given that one-third of the world population is estimated to be latently infected with TB, there is a huge reservoir for the development of future TB disease [6].

As long as a *M. tuberculosis* reservoir exists in individuals with LTBI, elimination of TB will not be feasible. Thus, the control of LTBI is an important step towards TB elimination. In addition to TB case detection and treatment, TB is controlled by identifying individuals who are latently infected with *M. tuberculosis* and offering them treatment that will prevent the development of TB disease, especially in high-income countries [7–9].

Several LTBI treatment regimens have shown effectivenesss [10]. However, adherence to these treatment regimens was sometimes low and differed between treatment regimens and populations [8, 11–18]. Numerous reasons for low adherence have been reported, such as (fear of) side effects of the treatment, lack of symptomatic disease and thus lack of motivation for taking preventive treatment, or low risk perception of progression to active TB [11, 17, 19].

Incorporating programmatic LTBI control into the national and EU/EEA strategies to fight TB is likely to be of value for all EU/EEA Member States. Therefore, the European Centre for Disease Prevention and Control (ECDC) aims to provide EU/EEA Member States and candidate countries with scientific advice and guidance on programmatic LTBI control. In order to collect the evidence base for developing the ECDC guidance a series of systematic reviews have been performed. This was done in collaboration with World Health Organization (WHO) who used the same evidence base for the development of the WHO guidelines on LTBI control [20] launched in early 2015. One important aspect for LTBI control is to ensure adherence to and completeness of the preventive treatment. Therefore, a systematic literature review was performed to assess initiation and completion rates of LTBI treatment and to identify determinants and interventions for adherence and completion, in the general and in specific populations with LTBI. In this article we present the results of the initiation and completion rates for recommended preventive treatments.

## Methods

A systematic literature review was performed according to a review protocol and following the Cochrane guidelines. The aim of the systematic review was to provide answers to the following research questions: 1) What is the LTBI treatment initiation rate and the completion rate for each recommended LTBI treatment regimen; 2) What are determinants of LTBI treatment initiation, adherence, and completion; 3) What are the interventions with demonstrated efficacy or effectiveness to improve LTBI treatment initiation, adherence and completion in individuals who are eligible for LTBI treatment. Due to the extensive results, the results for review question 1 are presented in this article and the results for review questions 2 and 3 will be presented separately.

### Eligibility criteria

PICO (Population-Intervention-Comparator-Outcome) questions were formulated based on the review questions (see Additional file 1: PICO questions). Only primary articles describing randomised controlled trials (RCTs), non-randomised prospective comparative studies of interventions, prospective longitudinal observational studies, and retrospective studies were included in this review. Systematic reviews were not included; however the reference lists of relevant systematic reviews were screened to find primary articles that were not found via our literature search. Studies in individuals eligible for LTBI treatment were considered relevant. Eligibility for LTBI treatment was defined as “being diagnosed with LTBI”. There was no required minimum study duration or number of subjects, except for studies in the general population diagnosed with LTBI that also presented data stratified for specific populations (e.g. case contacts, immigrants etc.). Since these studies were primarily aimed at the total population, and sampling strategies were applied accordingly, data for the specific populations were only extracted when such a population consisted of at least 30 subjects. Studies were considered to be conducted in the general population when they did not specifically focus on certain risk groups. For an article to be included in the review, baseline data (e.g. population characteristics) must be presented, LTBI had to be defined in the study (e.g. as “positive tuberculin skin tests (greater of equal 10 mm) and negative chest radiographs”) and the LTBI treatment regimen had to be specified, and for studies presenting completion rates, a definition for “completion” had to be provided. Studies did not have to apply a specific definition for LTBI or completion to be included. Adherence rates that met the definition of “completion” (e.g. “full adherence” or “adherence for nine months”) were interpreted as completion rates. If individuals whose completion status was pending by the end of a study were included in the completion rate, the rate was recalculated to exclude these individuals. Studies that included only case contacts who received chemoprophylaxis irrespective of whether or not LTBI was diagnosed were excluded from this review.

### Information sources and search strategy

We searched the databases PubMed and Embase. Search strings were composed for 1) LTBI, 2) LTBI treatment, and 3) initiation, adherence, completion and implementation. A fourth search string was composed to exclude animal studies (see Additional file 2: Search strings). No geographical, time, or language limits were applied, however only full-text articles in English, French, Spanish, German, and Dutch were reviewed. The search was carried out on February 3^rd^, 2014 for all literature published up to that date. Output from the searched databases was exported to Endnote version X4.0.2.

### Study selection

Articles were selected by a three-step selection procedure based on 1) screening of title and abstract, 2) screening of full-text article, and 3) final screening during the data-extraction phase. One-hundred percent of the title and abstract selection and critical appraisal of the full-text articles was done in duplicate by two independent researchers; the results were compared and discussed and any doubts were resolved by a third researcher.

### Risk of bias assessment

The risk of bias of each included full-text article was assessed with standardised, study-design specific, quality appraisal forms following the risk of bias assessment proposed by the Cochrane Collaboration [21]. A few additional aspects, not mentioned in the Cochrane Collaboration, were considered when evaluating the quality of the articles, i.e. the adequacy of recall assessment and reporting, whether confidence intervals were provided, and for retrospective studies, the adequacy of the method of retrospective selection of the population. Each aspect was evaluated as high risk of bias, moderate or unclear risk of bias, or low risk of bias. Because of the descriptive nature of the review question, risk of bias was only assessed for aspects of the individual studies, without providing an overall level of quality for each individual study. As review question 1 does not deal with the effects of health interventions and treatment and populations vary widely between studies, risk of bias was also not assessed across the evidence base per outcome.

### Data extraction

Evidence tables were compiled by two researchers and reviewed by a third researcher. The data extraction was done in duplicate for 15 % of the included articles, no major differences were found. Evidence tables were created for different populations with LTBI: 1) general population (primarily unselected individuals with LTBI at clinics), 2) case contacts, 3) healthcare workers, 4) the homeless, 5) people who inject drugs (PWID), 6) human immunodeficiency virus (HIV)-infected individuals, 7) inmates, 8) immigrants, and 9) patients with comorbidities, e.g. patients with rheumatoid arthritis or patients with hematologic malignancies. The definitions of completion were those used in the individual studies; there were differences in definitions between studies. Study results were sorted by study design and split by duration of the LTBI treatment regimen, i.e. short (≤four months), long (>four months), or short and long combined when no data were presented for short and long LTBI treatment separately.

### Synthesis of results

Meta-analysis is performed in accordance with GRADE methodology and results are reported in accordance with the Preferred Reporting Items for Systematic Reviews and Meta-Analyses (PRISMA) statement. For data visualisation, forest plots of initiation and completion rates were created for the identified populations in Excel 2010 [22]. The MetaXL 2.1 add-in in Excel was used to calculate 95 % confidence intervals around initiation and completion rates. We planned to calculate pooled rates, however this was not done for the first review question, because of the large heterogeneity of the included articles.

## Results

### Results of the review process

**Fig. 1:**
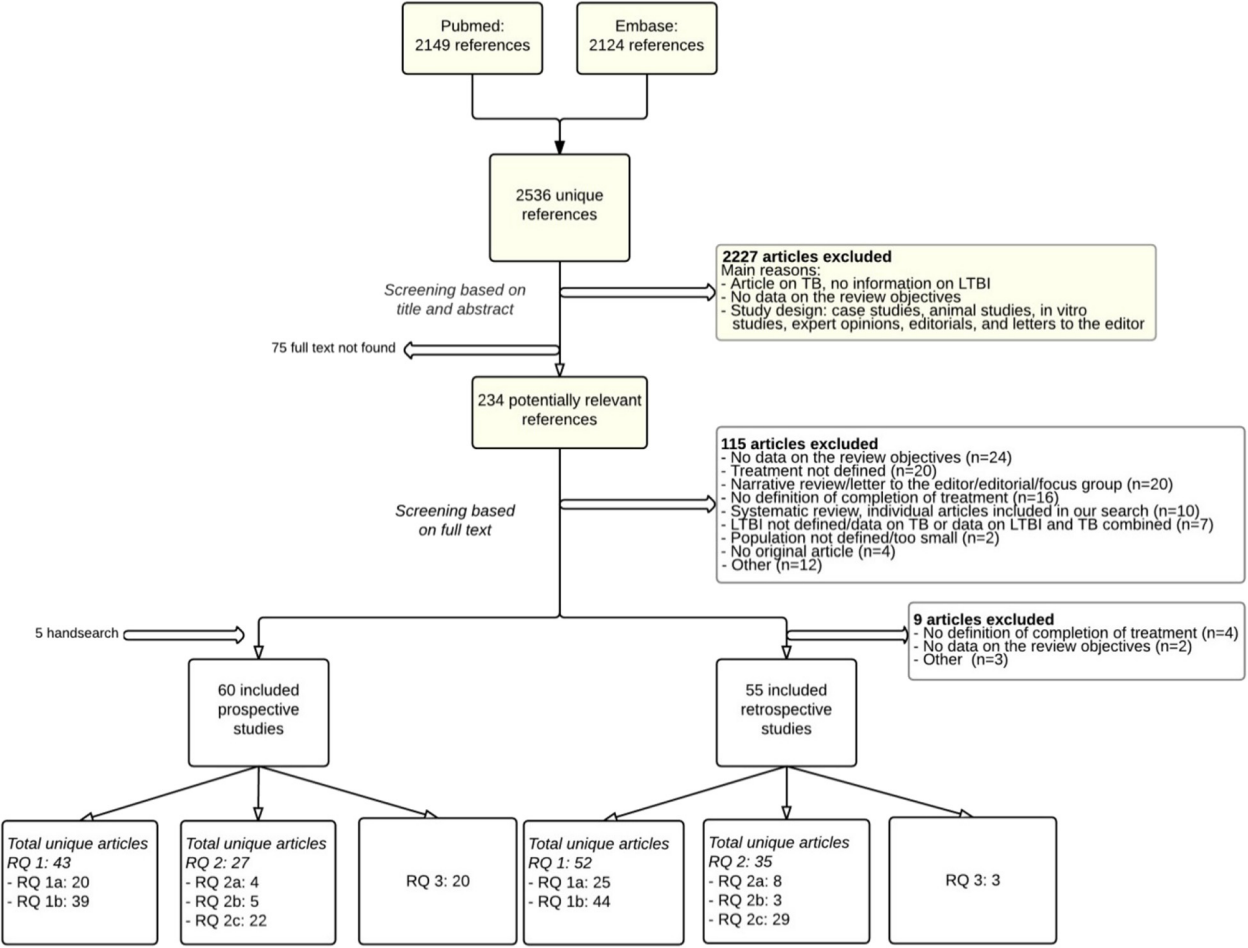
Flow chart of selection process. RQ: review question. LTBI: latent tuberculosis infection; TB: tuberculosis. Review question 1a: What is the initiation rate for each recommended LTBI treatment regimen?; Review question 1b: What is the completion rate for each recommended LTBI treatment regimen?; Review question 2a: What are the determinants of LTBI treatment initiation?; Review question 2b: What are the determinants of LTBI treatment adherence?; Review question 2c: What are the determinants of LTBI treatment completion?; Review question 3: In individuals who are eligible for LTBI treatment, what are the interventions with demonstrated efficacy or effectiveness to improve LTBI treatment initiation, adherence and completion?

The search resulted in 2536 unique hits; 115 relevant articles were included for all review questions (see Fig. 1). For review question 1, a total of 95 unique articles were found, including 43 prospective studies and 52 retrospective studies. Of these articles, 45 provided information on initiation rates of LTBI treatment regimens and 83 on completion rates. An overview of the study characteristics, initiation and/or completion rates, and the quality aspects of the risk of bias assessment of the included studies are presented in Additional file 3: Study characteristics, outcomes, and quality aspects.

### Initiation and completion rates

Most study populations in the included articles consisted of individuals from the general population diagnosed with LTBI, case contacts or immigrants with LTBI (Table 1; Additional file 3: Study characteristics, outcomes, and quality aspects). In prospective studies, initiation and completion rates were most often presented for long treatment regimens and in retrospective studies rates were most frequently reported for either long treatment regimens or for short and long treatment regimens combined.

**Table 1:** Number of included studies and ranges of initiation and completion rates per study population

Study population	Short treatment regimens		Long treatment regimens		Short/long treatment regimens combined		
	*n*	Range rates (%)	*n*	Range rates (%)	*n*	Range rates (%)	*N*
Initiation rates							
Prospective studies (*n* = 20 unique articles; some present data for more than one population and more than one treatment regimen)							
General	1 [23]	86	2 [18, 24]	44–99	1 [25]	26	4
Case contacts	0	–	3 [26–28]	40–85	1 [25]	53	4
Healthcare workers	1 [19]	98	0	–	0	–	1
Homeless	0	–	2 [29, 30]	76–90	1 [25]	34	3
PWID	0	–	3 [31–33]	52–91	0	–	3
HIV infected	0	–	2 [34, 35]	90–92	1 [36]	91	3
Inmates	0	–	1 [37]	65	0	–	1
Immigrants	0	–	3 [38–40]	77–97	1 [25]	23	4
Patients with comorbidities	0	–	0	–	0	–	0
Retrospective studies (*n* = 25 unique articles; some present data for more than one population and more than one treatment regimen)							
General	0	–	4 [89–92]	82–98	5 [16, 78, 93–95]	53–83	9
Case contacts	0	–	1 [96]	81	5 [13, 78, 97–99]	74–95	6
Healthcare workers	0	–	1 [100]	92	4 [78, 94, 101, 102]	47–89	5
Homeless	0	–	0	–	0	–	0
PWID	0	–	1 [103]	56	0	–	1
HIV infected	0	–	1 [104]	67	0	–	1
Inmates	0	–	0	–	2 [80, 105]	7–90	2
Immigrants	0	–	2 [106, 107]	78–84	3 [16, 93, 94]	57–82	5
Patients with comorbidities	1 [108]	93	1 [109]	82	0	–	2
Completion rates							
Prospective studies (*n* = 39 unique articles; some present data for more than one population and more than one treatment regimen)							
General	10 [12, 23, 25, 41, 42, 46, 47, 49, 50, 60]	61–95	10 [12, 18, 24, 25, 41, 42, 46, 47, 50, 57]	46–76	0	–	13
Case contacts	2 [51, 52]	71–82	4 [26, 27, 51, 52]	53–73	2 [12, 25]	60–64	6
Healthcare workers	0	–	1 [18]	44	0	–	1
Homeless	1 [60]	71	2 [18, 30]	25–33	1 [25]	44	4
PWID	0	–	4 [31, 32, 56, 58]	38–89	0	–	4
HIV infected	6 [36, 44, 48]	62–95	8 [18, 34–36, 44, 48, 54, 55]	55–89	0	–	8
Inmates	1 [53]	48	3 [37, 53, 62]	4–38	0	–	3
Immigrants	2 [43, 59]	72–80	8 [38–40, 43, 45, 57]	7–83	2 [12, 25]	61–79	9
Patients with comorbidities	1 [61]	87	0	–	0	–	1
Retrospective studies (*n* = 44 unique articles; some present data for more than one population and more than one treatment regimen)							
General	14 [14–17, 65–67, 78, 79, 81–83, 110, 111]	56–93	23 [14–16, 64–67, 78, 79, 81–83, 89–92, 112–118]	39–96	1 [93]	54	27
Case contacts	3 [13, 14, 17]	63–69	4 [13, 14, 113, 116]	56–78	5 [78, 82, 97, 98, 119]	48–81	10
Healthcare workers	0	–	2 [100, 113]	17–75	3 [15, 78, 101]	40–79	5
Homeless	1 [120]	44	1 [64]	23	0	–	2
PWID	0	–	1 [103]	55	0	–	1
HIV infected	0	–	2 [14, 104]	55–66	0	–	2
Inmates	4 [80, 120]	48–100	2 [80, 121]	23–68	0	–	3
Immigrants	4 [14, 16, 17, 122]	60–85	11 [14, 16, 64, 89, 91, 92, 106, 113, 116, 123, 124]	38–86	5 [15, 65, 79, 82, 93]	53–69	18
Patients with comorbidities	1 [108]	92	1 [125]	75	0	–	2

*HIV* human immunodeficiency virus, *n* number of included studies, *N* total number of included studies, *PWID* people who inject drugs

#### Initiation rates

**Fig. 2:**
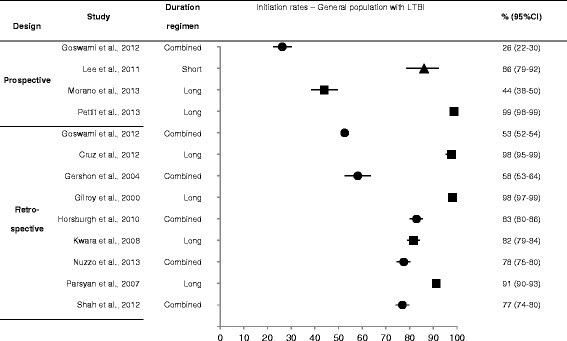
Forest plots with ranges of initiation rates in the general population diagnosed with LTBI. LTBI: latent tuberculosis infection; Circle: combined, square: long, triangle: short

Overall, twenty prospective studies [18, 19, 23–40] that reported initiation rates were found (Table 1; Additional file 3: Study characteristics, outcomes, and quality aspects). Initiation rates varied considerably among populations with LTBI, ranging from 26 to 99 % in the general population (four studies [18, 23–25]) (Fig. 2), from 40 to 85 % in case contacts (four studies [25–28]), from 34 to 90 % in the homeless (three studies [25, 29, 30]), from 52 to 91 % in PWID (three studies [31–33]), from 90 to 92 % in HIV-infected individuals (three studies [34–36]), and from 23 to 97 % in immigrants (four studies [25, 38–40]) (Additional file 3: Study characteristics, outcomes, and quality aspects). Only one prospective study each reported an initiation rate in healthcare workers or inmates, the initiation rates for these groups were 98 % [19] and 65 % [37], respectively. No prospective studies that reported initiation rates were found for patients with comorbidities.

Twenty-five retrospective studies that reported initiation rates, mostly based on medical records, were found (Table 1; Additional file 3: Study characteristics, outcomes, and quality aspects). There was considerable overlap in the ranges of initiation rates reported in prospective studies and retrospective studies.

#### Completion rates

In total, 39 prospective studies reporting completion rates were identified [12, 18, 23–27, 30–32, 34–62]. Completion rates ranged from 46 to 95 % in the general population (thirteen studies [12, 18, 23–25, 41, 42, 46, 47, 49, 50, 57, 60]), from 53 to 82 % in case contacts (six studies [12, 25–27, 51, 52]), from 25 to 71 % in the homeless (four studies [18, 25, 30, 60]), from 38 to 89 % in PWID (four studies [31, 32, 56, 58]), from 55 to 95 % in HIV-infected individuals (eight studies [18, 34–36, 44, 48, 54, 55]), from 4 to 48 % in inmates (three studies [37, 53, 63]), and from 7 to 83 % in immigrants (nine studies [12, 25, 38–40, 43, 45, 57, 59]). Only one study reported a completion rate in healthcare workers and patients with comorbidities, the completion rates for these groups were 44 % [18] and 87 % [61], respectively. In addition, 44 retrospective studies reporting completion rates were found (Table 1; Additional file 3: Study characteristics, outcomes, and quality aspects). Within-study comparisons show that the homeless had lower completion rates than other populations [18, 25, 64]. As for initiation rates, considerable overlap in the ranges of completion rates reported in prospective studies and retrospective studies appears to exist.

#### Short versus long treatment regimens

**Fig. 3:**
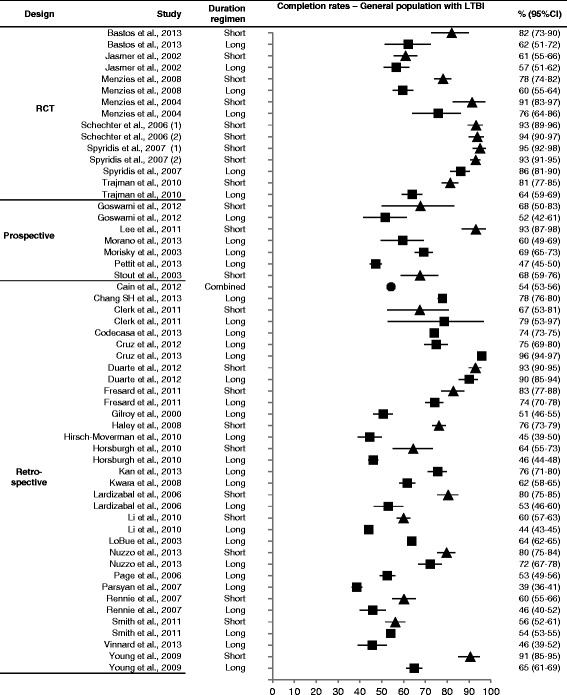
Forest plot with completion rates in the general population diagnosed with LTBI. LTBI: latent tuberculosis infection; Circle: combined, square: long, triangle: short

In most of the 27 studies that presented separate completion rates for patients that received short treatment regimens and for patients that received long treatment regimens, completion rates appear to be higher for the short treatment group (Fig. 3; Additional file 4: Forest plots). However, an exception to this observation was Clerk et al., who showed higher completion rates for long treatment regimens than shorter regimens (67 % vs 79 %) [65], and a small number of other studies that showed very similar completion rates between the two treatment groups [48, 52, 66, 67] in various populations with LTBI. Due to large heterogeneity across studies and the fact that most studies were not designed to specifically assess the effect of treatment duration on completion, no pooled analysis was performed to determine if there was a statistically significant association between treatment duration and completion rates.

## Discussion

This is, to our knowledge, the first systematic review to comprehensively explore both initiation and completion rates of LTBI treatment worldwide. In total, evidence from 95 studies has been reviewed. Forty-five studies reported on initiation rates and 83 on completion rates, covering nine different population types with LTBI. There was wide variation in initiation and completion rates; the initiation rates ranged from 7 to 99 % and the completion rates ranged from 4 to 100 % across the different population groups. These rates should be interpreted taking into account the variety of treatment options (i.e. choice of regimen, duration, self-administered or observed).

### Risk groups

The population for which most studies were identified was the general population diagnosed with LTBI. Though this population consisted primarily of unselected individuals with LTBI at clinics, it was very diverse, mainly due to the varying proportion of specific populations, such as immigrants, across clinics. Generally, two types of risk groups could be distinguished: groups with higher risk of TB infection, but without an increased risk of progression to TB (e.g. health care workers, inmates, and the homeless) [68–70] and groups with LTBI who are at higher risk of progression to active TB (e.g. HIV-infected individuals, patients with comorbidities) [71–74]. Case contacts appear to have both a higher risk of TB infection and a higher risk of progression to active TB due to recent infection [68, 75]. The initiation rates and completion rates of LTBI treatment appeared slightly higher in the groups with higher risk of progression of LTBI to active TB than in the groups with higher risk of TB infection. Care has to be taken when comparing findings between studies, due to differences within populations, in setting and in methodology.

Hirsch-Moverman et al. [11] reviewed studies in the United States and Canada published between 1997 and 2007 and presented completion rates of LTBI treatment that are in line with the results found in our review*.* Overall, 20 of the 60 studies that were included in their review were also included in ours; differences are due to the fact that we did not include studies that did not provide a definition for completion, did not specify the type of treatment or did not define LTBI.

Al-Darraji et al. [76] reviewed completion rates of treatment with isoniazid in inmates with LTBI, from studies published between 1966 and January 2011*.* Four of the sixteen studies they included were also included in our review. Both the presented completion rate of isoniazid treatment during incarceration and after release from jail appeared slightly higher than the completion rates of long LTBI treatment regimens in inmates found in our review.

### Short and long treatment regimens

Short LTBI treatment most frequently consisted of four months of rifampicin or two months of rifampicin and pyrazinamide, though other combinations of rifampicin, isoniazid, rifabutin and pyrazinamide were also administered for two to four months. Currently, the combination of rifampicin with pyrazinamide is not generally offered to persons with LTBI as it has been associated with hepatotoxicity [77]. Long LTBI treatment consisted of isoniazid regimens, usually for six or nine months and often for twelve months in the case of concurrent HIV-infection. Only three studies presented initiation rates for short treatment regimens, therefore initiation rates between short and long treatment regimens could not be compared.

A considerable number of prospective articles presented completion rates for both short and long treatment regimens, especially in the general population diagnosed with LTBI and in HIV-infected individuals with LTBI. Most results of within-study comparisons of short and long treatments [13–16, 78–83] indicated higher completion rates for shorter regimens, although in two studies rates were comparable for short and long treatment regimens [66, 67]. In one study lower completion rates were reported for short treatment than for longer treatments [65] due to adverse effects. It should be kept in mind that the type of drugs used in short and long LTBI treatment regimens varied considerably between studies and within populations.

Hirsch-Moverman et al. [11] also reviewed data on completion rates of short (rifampicin or rifampicin in combination with pyrazinamide) and long (isoniazid) treatment regimens within studies and found a higher percentage of completion among short treatments in all included studies, although the difference was not always significant*.* Ziakas et al. [84] systematically reviewed the literature for studies that compared four months of rifampicin with nine months of isoniazid, published up to July 2009, and included four studies in a meta-analysis. These four studies were also included in this review. They concluded that four months of rifampicin was associated with a significant reduction in the risk of non-completion [84]. Sharma et al. [85] conducted a systematic review on RCTs worldwide among HIV-negative people at risk of active TB that compared rifampicin monotherapy or rifamycin-combination therapy with isoniazid monotherapy. Their meta-analysis of five trials, of which two were included in our review, compared rifampicin (three or four months) with isoniazid (six months) and showed that significantly more people completed the shorter course. Two more of their meta-analyses compared isoniazid in combination with rifampicin (three months; two trials) or rifampicin in combination with pyrazinamide (two months; three trials, one of which was included in our review) with isoniazid (six months) and found that there was no difference in completion rates [85].

By comparing completion rates between short and long LTBI treatment we do not take into account any effect of the treatment regimen on completion rate, e.g. due to different side effect patterns. The effect of different treatment regimens for preventing active TB, and thus indirectly taking into account completion, has been compared by Stagg et al. [10] and was part of the same evidence base collection as our systematic review.

### Limitations

Studies without a definition for “completion” were excluded from this review. Still, the definition of completion varied considerably between studies, and this heterogeneity complicated comparison of completion rates between studies. Definitions used varied from “completed four months of rifampicin” [81] to “picked up nine months of isoniazid within twelve months” [25] and “took at least 80 % of the prescribed medication within twenty weeks” [15]. Also, the quality of the definitions of “completion” varied throughout the included studies and the distinction between definitions for adherence and completion was not always obvious. Besides, the quality of the definition of “completion” is closely linked to the way treatment adherence is assessed, which varied from self-reported adherence (least reliable) [35], dispense of medication [58], pill counts [52], urine tests [43], to Directly Observed Treatment (DOT; most reliable) [62]. Furthermore, it was not always clear whether and to what extent the classification of a patient as having completed treatment was influenced by the time that a patient needed to take all the doses. Finally, some patients may have discontinued treatment due to toxicity. In our analysis, this is included in non completion. Discontinuation because of side effects will influence the effectiveness of latent TB infection treatment but it will need other efforts than voluntary discontinuation of treatment.

In addition, rate calculations varied across included studies; different rules were applied for in- and exclusion into the denominator to calculate the initiation and/or completion rates. All these differences between studies and unclarities hampered the analysis.

Within our review, no distinction was made between studies that looked at a priori defined risk groups versus studies that identified risk factors in a non-selected group of individuals with LTBI. Also, population characteristic or settings of risk groups may vary between studies, for example immigrants in the United States might differ from immigrants in Europe with respect to their origin [86, 87]. In addition, it should be noted that individuals with LTBI might belong to multiple risk groups, as overlap between the groups exists; however, most studies did not specify if there was any overlap between risk groups.

## Conclusions

The goal of LTBI treatment is to eradicate *M. tuberculosis* from the body and to reduce the risk of progression to active TB disease [88]. Only treatment can clear the bacteria from the body, and therefore individuals with LTBI who do not initiate treatment contribute to the persistence of the TB reservoir. Clinical benefit to the individual with LTBI and the success of control programs is subject to completion of LTBI treatment.

This systematic review found that initiation and completion rates for LTBI treatment regimens were frequently suboptimal and varied greatly within and across different populations. Population groups with a higher risk of TB infection, but without an increased risk of progression to TB (e.g. health care workers, inmates, and the homeless) seemed at slightly higher risk of low initiation and completion rates. However, the limited number of studies found for certain LTBI treatment regimens and study populations, and the variation in types of risk populations, settings, treatment regimens and study methodologies in the included studies make it difficult to draw firm conclusions.

Considering the sub-optimal rates of completion, implementing interventions to improve the adherence and completion rates in groups most at risk of developing active TB seems relevant to make LTBI treatment more effective and decrease the spread of TB.

## Ethics approval and consent to participate

Not applicable.

## Consent for publication

Not applicable.

## Availability of data and materials

The datasets supporting the conclusions of this article are included within the article and its additional files.

## Additional files

Additional file 1: PICO questions. (DOC 45 kb) Additional file 2: Search strings. (DOC 29 kb) Additional file 3: Study characteristics, outcomes, and quality aspects of risk of bias assessment of articles on initiation and completion rates of LTBI treatment regimens. (DOC 680 kb) Additional file 4: Forest plots. (DOC 396 kb)

## Abbreviations

ECDC: European Centre for Disease Prevention and Control
EU/EEA: European Union and European Economic Area
HIV: human immunodeficiency virus
LTBI: latent TB infection
PICO: Population-Intervention-Comparator-Outcome
PWID: people who inject drugs
RCTs: randomised controlled trials
TB: tuberculosis
WHO: World Health Organization
