# Supplementary material for: Initiation and completion rates for latent tuberculosis infection treatment: a systematic review
Source: BMC Infect Dis. 2016 May 17;16:204. doi: 10.1186/s12879-016-1550-y (PMC4869320; doi:10.1186/s12879-016-1550-y)
Supplement: Additional file 1: — PICO questions. (DOC 45 kb) [file 12879_2016_1550_MOESM1_ESM.doc]

# Additional file 1: PICO questions

The following PICO questions were used to define the search string:

| 1a | What is the LTBI treatment initiation rate for each recommended LTBI treatment regimen |
| --- | --- |
| P | Individuals with indication for LTBI treatment |
| I | LTBI treatment regimens |
| C | Not applicable |
| O | Proportion of eligible individuals who initiate treatment |

| 1b | What is the completion rate for each recommended LTBI treatment regimen |
| --- | --- |
| P | Individuals who initiate LTBI treatment |
| I | LTBI treatment regimens |
| C | Not applicable |
| O | Proportion of eligible individuals who completed treatment |

| 2a | What are the determinants of LTBI treatment initiation? |
| --- | --- |
| P | Individuals with indication for LTBI treatment |
| I | Individuals who initiate LTBI treatment |
| C | Individuals who do not initiate LTBI treatment |
| O | Determinants of treatment initiation |

| 2b | What are the determinants of LTBI treatment adherence? |
| --- | --- |
| P | Individuals who initiate LTBI treatment |
| I | Individuals who adhere to LTBI treatment |
| C | Individuals who do not adhere to LTBI treatment |
| O | Determinants of treatment adherence |

| 2c | What are the determinants of LTBI treatment completion? |
| --- | --- |
| P | Individuals who initiate LTBI treatment |
| I | Individuals who complete LTBI treatment |
| C | Individuals who do not complete LTBI treatment |
| O | Determinants of treatment completion |

| 3 | In individuals who are eligible for LTBI treatment, what are the interventions with demonstrated efficacy or effectiveness to improve LTBI treatment initiation, adherence and completion? |
| --- | --- |
| P | Individuals with indication for LTBI treatment |
| I | Individuals with LTBI treatment receiving the intervention to improve LTBI treatment initiation, adherence and/or completion |
| C | Individuals with LTBI treatment not receiving the intervention to improve LTBI treatment initiation, adherence and/or completion |
| O | Proportion of eligible individuals who initiated, adhered and/or completed treatment  Other outcomes for efficacy or effectiveness, acceptability and feasibility of the intervention |
