# Supplementary material for: Initiation and completion rates for latent tuberculosis infection treatment: a systematic review
Source: BMC Infect Dis. 2016 May 17;16:204. doi: 10.1186/s12879-016-1550-y (PMC4869320; doi:10.1186/s12879-016-1550-y)
Supplement: Additional file 2: — Search strings. (DOC 29 kb) [file 12879_2016_1550_MOESM2_ESM.doc]

# Additional file 2: Search strings

**PubMed**

*#A Search string for LTBI:*

“latent tuberculosis” [Mesh] OR “latent tuberculosis” [tiab] OR LTB [tiab] OR LTBI [tiab] OR ((laten* [tiab] OR dorman* [tiab]) AND (TB [tiab] OR tuberc* [tiab]))

*#B Search string for LTBI treatment:*

"therapeutics" [Mesh] OR "therapy" [Subheading] OR "treatment outcome" [Mesh] OR "primary prevention" [Mesh] OR "secondary prevention" [Mesh] OR “prevention and control” [Subheading] OR treatment* [tiab] OR therapy [tiab] OR therapies [tiab] OR therapeutics [tiab] OR prevent* [tiab] OR management [tiab] OR “antibiotic prophylaxis” [Mesh] OR “chemoprevention“ [Mesh] OR prophyla* [tiab] OR chemoprophylaxis [tiab] OR DOT [tiab] OR DOTS [tiab] OR "isoniazid" [Mesh] OR isoniazid [tiab] OR INH [tiab] OR IPT [tiab] OR "rifapentine" [Supplementary Concept] OR rifapentine [tiab] OR RPT [tiab] OR "rifampin" [Mesh] OR rifampin [tiab] OR RIF [tiab] OR rifampicin [tiab] OR ethambutol [tiab] OR EMB [tiab] OR ethionamide [tiab] OR ETH [tiab] OR pyrazinamide [tiab] OR PZA [tiab] OR fluroquinolones [tiab] OR FLQ [tiab] OR moxifloxacin [tiab] OR levofloxacin [tiab] OR gatifloxacin [tiab]

*#C Search string for adherence, initiation, completion, and implementation:*

"attitude" [Mesh] OR adher* [tiab] OR “medication adherence” [Mesh] OR “guideline adherence” [mesh] OR “patient compliance” [mesh] OR complian* [tiab] OR comply* [tiab] OR accordance [tiab] OR according [tiab] OR agreement [tiab] OR “withholding treatment” [mesh] OR initiat* [tiab] OR start [tiab] OR commenc* [tiab] OR begin* [tiab] OR introduc* [tiab] OR enroll* [tiab] OR complet* [tiab] OR finaliz* [tiab] OR finalis* [tiab] OR fulfill* [tiab] OR ending [tiab] OR finish* [tiab] OR terminat* [tiab] OR accomplish* [tiab] OR realiz* [tiab] OR realis* [tiab] OR attain* [tiab] OR implement* [tiab] OR apply* [tiab] OR application* [tiab] OR “medication therapy management” [mesh]

*#D Search string for animal studies:*

Animals [Mesh] NOT (Humans [Mesh] AND Animals [Mesh])

*Search:*

((#A AND #B AND #C) NOT #D)

**EMBASE**

*#A Search string for LTBI:*

“latent tuberculosis”/exp OR (tuberculosis/exp AND 'latent period'/de) OR (LTB OR LTBI OR ((laten* OR dorman*)  AND (TB OR tuberc*))):ab,ti

*#B Search string for LTBI treatment:*

“therapy”/exp OR therapy:lnk OR “treatment outcome”/exp OR “primary prevention”/exp OR "prevention and control"/de OR “secondary prevention”/exp OR prevention:lnk OR “antibiotic prophylaxis”/exp OR “chemoprophylaxis”/exp OR “isoniazid”/exp OR “rifampicin”/exp OR (treatment* OR therap* OR prevent* OR management OR control OR prophyla* OR chemoprophyla* OR DOT OR DOTS OR isoniazid OR INH OR IPT OR rifapentine OR RPT OR rifampin OR RIF OR ethambutol OR EMB OR ethionamide OR ETH OR pyrazinamide OR PZA OR fluroquinolones OR FLQ OR moxifloxacin OR levofloxacin OR gatifloxacin):ab,ti

*#C search string for adherence, initiation, completion and implementation:*

“medication compliance”/exp OR “patient attitude”/de OR “treatment withdrawal”/exp OR “medication therapy management”/exp OR "treatment refusal"/exp OR "refusal to participate"/de OR (adher* OR complian* OR comply* OR accordance OR according OR agreement OR Initiat* OR start* OR commenc* OR begin OR introduc* OR enroll* OR complet* OR finaliz* OR finalis* OR fulfill* OR ending OR finish* OR terminat* OR accomplish* OR realiz* OR realis* OR attain* OR implement* OR apply* OR application* OR refus* OR attitud*):ab,ti

*#D Search string for animal studies:*

(“animal”/exp NOT “human”/exp)

*Search:*

((#A AND #B AND #C) NOT #D)
