# Supplementary material for: Initiation and completion rates for latent tuberculosis infection treatment: a systematic review
Source: BMC Infect Dis. 2016 May 17;16:204. doi: 10.1186/s12879-016-1550-y (PMC4869320; doi:10.1186/s12879-016-1550-y)
Supplement: Additional file 3: — Study characteristics, outcomes, and quality aspects of risk of bias assessment of articles on initiation and completion rates of LTBI treatment regimens. (DOC 680 kb) [file 12879_2016_1550_MOESM3_ESM.doc]

# Additional file 3: Study characteristics, outcomes, and quality aspects of risk of bias assessment of articles on initiation and completion rates of LTBI treatment regimens

| **Reference, country** | **Study design** | **Study population**  **(sample size)** | **Treatment, initiation rate (IR) or completion rate (CR)**^f^ | | | **Quality aspects risk of bias assessment of each study^g^** |
| --- | --- | --- | --- | --- | --- | --- |
|  |  |  | **Short** | **Long** | **Short&long combined** |  |
| **Randomized controlled trials** | | | | | | |
| Bastos et al. 2013 Brazil | International multicentre clinical trial (parent trial) | General population  (n=160) | 4R, SAT  CR: 64/78 = 82% | 9H, SAT  CR: 51/82 = 62% | - | Low: 5, 6, 8  Moderate: 1, 2, 3, 4, 7  High: - |
| Jasmer et al. 2002 USA | Multicentre, prospective open-label clinical trial | General population  (n=589) | 2RZ, SAT  CR: 187/307 = 61% | 6H, SAT  CR: 160/282 = 57% | - | Low: 5, 8  Moderate: 2, 3, 6  High: 1, 4, 7 |
| Jiménez-Fuentez et al. 2013 Spain | RCT | Immigrants  (n=590) | 3HR, SAT  CR: 213/296 = 72% | 6H, SAT  CR: 154/294 = 52% | - | Low: 1, 6, 7, 8, 5  Moderate: 2, 3  High: 4 |
| Matteelli et al. 1999 Italy | Randomised, comparative open label pilot study | HIV-infected individuals  (n=44) | 3H+RFB, SAT  CR: 26/30 = 87% | 6H, SAT  CR: 10/14 = 71% | - | Low: 4, 5, 6, 7  Moderate: 1, 2, 8^A^  High: 3 |
| Matteelli et al. 2000 Italy | Multicenter, prospective randomised open-label study | Immigrants  (n=208) | - | 6H, SAT/DOT  CR H (DOT): 60/82 = 7%  CR H (SAT, twice weekly): 19/73 = 26%  CR H (SAT, daily): 22/53 = 41% | - | Low: 1, 4, 5, 6, 7, 8  Moderate: 2  High: 3 |
| Menzies et al. 2004 Canada | Open-label RCT | General population  (n=116) | 4R, SAT  CR: 53/58 = 91% | 9H, SAT  CR: 44/58 = 76% | - | Low: 1, 4, 5, 6, 7  Moderate: 2  High: 3, 8^B^ |
| Menzies et al. 2008 Brazil, Canada, Saudi Arabia | Multicentre, randomised open-label trial | General population  (n= 847) | 4R, SAT  CR: 328/420 = 78% | 9H, SAT  CR: 255/427 = 60% | - | Low: 1, 4, 5, 6, 7, 8  Moderate: 2  High: 3 |
| Rivero et al. 2007 Spain | Multicenter RCT | HIV-infected individuals  (n=308) | 3R/2RZ, SAT  CR: 126/201 = 63% | 6H, SAT  CR: 69/107 = 64% | - | Low: 1, 4, 5, 6, 8  Moderate: 2  High: 3, 7 |
| Schechter et al. 2006 Brazil | Controlled clinical trial | General population  (n=399) | 2RZ/3H+RPT. DOT  CR (2RZ): 192/206 = 93%  CR (3H+RPT): 181/193 = 94% | - | - | Low: 1, 2, 4, 5, 6  Moderate: 3  High: 7, 8^M^ |
| Spyridis et al. 2007 Greece | RCT | General population  (n=926) | 3HR/4HR, SAT  CR (3HR): 209/220 = 95%  CR (4HR): 441/474 = 93% | 9H, SAT  CR: 200/232 = 86% | - | Low: 1, 4, 5, 6, 7, 8  Moderate: 2  High: 3 |
| Sterling et al. 2011 Brazil, Canada, Spain, USA | Prospective open-label randomised non-inferiority trial | Case contacts  (n=7731) | 3H+RPT, DOT  CR: 3273/3986 = 82% | 9H, SAT  CR: 2585/3745 = 69% | - | Low: 1, 3, 4, 5, 8  Moderate: 2  High: 3, 7 |
| Tortajada et al. 2005 Spain | Multicentered randomised, comparative and prospective  trial | Case contacts (n=352) | 2RZ, SAT  CR: 109/153 = 71% | 6H, SAT  CR: 145/199 = 73% | - | Low: 1, 5, 6, 7, 8  Moderate: 2  High: 3, 4 |
| Trajman et al. 2010 Canada, Brazil, Saudi Arabia | RCT | Case contacts  (n=259) | - | - | 4R/9H, SAT  CR: 167/259 = 64% | Low: 1, 4, 6, 7, 8  Moderate: 2  High: 3, 5 |
|  |  | Immigrants  (n=513) | - | - | 4R/9H, SAT  CR: 403/513 = 79% |  |
| **Prospective observational studies** | | | | | | |
| Bark et al. 2010  Uganda | Multicenter prospective cohort study | HIV-infected individuals  (n=48) | - | 9H, SAT  IR: 44/48 = 92%  CR: 39/44 = 89% | - | Low: -  Moderate: 9  High: 10, 11^A^ |
| Bock et al. 1999  USA | Prospective cohort study | Homeless individuals  (n=409) | - | 6 or 9H/R, SAT  IR: 310/409 = 76% | - | Low: 11  Moderate: -  High: 10 |
| Bock et al. 2001  USA | Prospective cohort study | Inmates  (n= 400) | 2RZ, DOT  CR: 81/168 = 48% | >6H, unknown  CR: 9/232 = 4% | - | Low: 9  Moderate: 11  High: 10 |
| Brassard et al. 2004  Canada | Prospective cohort study | Homeless individuals  (n=25) | - | 6H, SAT  IR: 13/25 = 52%  CR: 5/13 = 38% | - | Low: 9  Moderate: -  High: 10, 11^A, C^ |
| Golub et al. 2009  South Africa | Prospective cohort study | HIV-infected individuals  (n=355) | - | 6H, SAT  CR: 209/355 = 59% | - | Low: 11  Moderate: -  High: 9, 10 |
| Goswami et al. 2012  USA | Prospective cohort study | General population  (n=496) | 4R, SAT  CR: 64/78 = 82% | 9H, SAT  CR: 51/99 = 52% | 4R/9H, SAT  IR: 130/496 = 26% | Low: 11  Moderate: -  High: 9, 10 |
|  |  | Case contacts (n=94) | - | - | 4R/9H, SAT  IR: 50/94 = 53%  CR: 30/50 = 60% |  |
|  |  | Homeless individuals  (n=53) | - | - | 4R/9H, SAT  IR: 18/53 = 34%  CR: 8/18 = 44% |  |
|  |  | Immigrants  (n=289) | - | - | 4R/9H, SAT  IR: 67/289 = 23%  CR: 41/67 = 61% |  |
| Hiransuthikul et al. 2005  Thailand | RCT, but only one arm described here | HIV-infected individuals  (n=181) | - | 6H, SAT  CR: 157/181 = 87% | - | Low: 9, 10  Moderate: -  High: 11^C^ |
| Lashley et al. 2007  USA | Intervention study | Homeless individuals  (n=30) | - | 9H, SAT  IR: 27/30 = 90%  CR: 9/27 = 33% | - | Low: -  Moderate: 11^A^  High: 9, 10 |
| Lee et al. 2012  Korea | Prospective study | General population  (n=101) | 4R, DOT  IR: 87/101 = 86%  CR: 81/87 = 94% | - | - | Low: 9, 10  Moderate: 11  High: - |
| Lorvick et al. 1999  USA | Single arm intervention | PWID  (n=27) | - | 6H, DOT  CR: 24/27 = 89% | - | Low: 9, 10  Moderate: 11^A^  High: - |
| Machado et al. 2009  Brazil | Prospective cohort study | Case contacts  (n=135) | - | 6H, SAT  IR: 101/135 = 75%  CR: 54/101 = 53% | - | Low: 11  Moderate: 9  High: 10 |
| Minodier et al. 2010 Canada | Prospective descriptive study | Immigrants  (n=545) | - | 6H/9H, SAT  IR: 573/645 = 89%  CR: 334/545 = 61% | - | Low: 9, 11  Moderate: -  High: 10 |
| MMWR. March 21, 2003  USA | Observational study  (case tracing) | Case contacts  (n=67) | - | 9H, DOT/SAT  IR: 57/67 = 85%  CR: 36/57 = 63% | - | Low: -  Moderate: 9  High: 10, 11^A, B^ |
| Morano et al. 2013  USA | Prospective cohort study | General population  (n= 307) | - | 9H, DOT/SAT  IR: 135/307 = 44%  CR (DOT): 56/94 = 60%  CR (SAT): 19/41 = 46% | - | Low: -  Moderate: 9  High: 10, 11 |
| Morisky et al. 2003  USA | Combination retrospective and prospective chart review | General population  (n= 478) | - | 6H, SAT  CR: 331/478 = 71% | - | Low: 9  Moderate: -  High: 10, 11 |
|  |  | Immigrants  (n=254) | - | 6H, SAT  CR: 162/254 = 64% | - |  |
| Narita et al. 2002  USA | Prospective cohort study | HIV-infected individuals  (n=176) | 2RZ/2RFB+Z, DOT  CR: 126/135 = 93% | 12H, SAT  CR: 17/25 = 68% | 2RZ/2RFB+Z/12H, SAT/DOT  IR: 160/176 = 91% | Low: 9, 11  Moderate: -  High: 10 |
| Nolan et al. 1997  USA | Prospective cohort study | Inmates  (n=744) | - | 9H, SAT/DOT  IR: 483/744 = 65%  CR: 115/301 = 38% | - | Low: 9, 11  Moderate: -  High: 10 |
| Oni et al. 2012  South Africa | Prospective study | HIV-infected individuals  (n=183) | - | 6H, SAT  IR: 164/183 = 90%  CR: 113/164 = 69% | - | Low: -  Moderate: 9  High: 10, 11 |
| Pettit et al. 2013 Canada, USA | Prospective cohort study | General population  (n=1323) | - | 6H/9H, SAT  IR: 1306/1323 = 99%  CR: 617/1306 = 47% | - | Low: 9, 11  Moderate: -  High: 10 |
|  |  | HCW (n=88) | - | 6H/9H, SAT  CR: 39/88 = 44% | - |  |
|  |  | Homeless individuals  (n=40) | - | 6H/9H, SAT  CR: 10/40 = 25% | - |  |
|  |  | HIV-infected individuals  (n=31) | - | 6H/9H, SAT  CR: 17/31 = 55% | - |  |
| Rutherford et al. 2013  Indonesia | Prospective cohort study | Case contacts  (n=15) | - | 6H, SAT  IR: 6/15 = 40% | - | Low: -  Moderate: -  High: 10, 11^A, B^ |
| Sadaphal et al. 2001  USA | Prospective study | PWID  (n=146) | - | 6H/12H, DOT  CR: 102/146 = 70% | - | Low: 9, 10,11  Moderate: -  High: - |
| Sarivalasis et al. 2013  Switzerland | Prospective cohort study | Immigrants  (n=75) | 4R, SAT  CR: 59/74 = 80%^a^ | - | - | Low: 9  Moderate: -  High: 10, 11^A^ |
| Scholten et al. 2003  USA | Prospective cohort study | PWID  (n=995) | - | 6H/12H, DOT  IR: 607/995 = 61%  CR: 259/607 = 43% | - | Low: 9, 10, 11  Moderate: -  High: - |
| Shukla et al. 2002  USA | Prospective cohort study | HCW  (n=404) | - | 6H, SAT  IR: 396/404 = 98% | - | Low: 11  Moderate: -  High: 10 |
| Snyder et al. 1999  USA | Prospective study | PWID  (n=417) | - | 6H/12H, DOT  IR: 378/417 = 91% | - | Low: 10, 11  Moderate: -  High: - |
| Stout et al. 2003  USA | Prospective cohort study | General population  (n=114) | 2RZ, SAT/DOT  CR: 77/114 = 68% | - | - | Low: -  Moderate: 9, 11  High: 10 |
|  |  | Homeless individuals  (n=69) | 2RZ, SAT/DOT  CR: 49/69 = 71% | - | - |  |
| Trauer et al. 2011  Australia | Prospective study | Immigrants  (n=121) | - | 9H, SAT  IR: 93/121 = 77%  CR: 41/93 = 44% | - | Low: 9, 11  Moderate: -  High: 10 |
| Valls et al. 2014  Spain | Open-label, single-arm intervention study | Patients with comorbidities^b^  (n=69) | 3HR, SAT  CR: 60/69 = 87% | - | - | Low: 9  Moderate: -  High: 10, 11^A^ |
| White et al. 2005  USA | Prospective cohort study | Inmates  (n=557) | - | 6H, SAT  CR: 176/557 = 32% | - | Low: 9  Moderate: 10  High: 11^C^ |
| Young et al. 2012  USA | Historical control study | Immigrants  (n=150) | - | 9H, SAT  IR: 146/150 = 97%  CR: 121/146 = 83% | - | Low: 9, 10, 11  Moderate: -  High: - |
| **Retrospective observational studies** | | | | | | |
| Ailinger et al. 1998  USA | Retrospective review of medical records | Immigrants  (n=65) | - | 6H, SAT  CR: 36/65 = 55% | - | Low: 14  Moderate: 13, 16^A^  High: 12, 15 |
| Ailinger et al. 2006  USA | Retrospective review of medical records | Immigrants  (n=53) | - | 9H, SAT  CR: 38/53 = 72% | - | Low: 12, 14  Moderate: 13, 15, 16^A^  High: - |
| Ailinger et al. 2007  USA | Retrospective review of medical records | Immigrants  (n=153) | - | 9H, SAT  IR: 129/153 = 84%  CR: 52/129 = 40% | - | Low: 12, 14, 16  Moderate: 15  High: 13 |
| Anger et al. 2012 USA | Retrospective review of medical records | Case contacts  (n=7597) | 4R, SAT  CR: 441/640 = 69% | 6H/9H, SAT  CR: 3201/5361 = 60% | 4R/6H/9H, SAT  IR: 6001/7597 = 79% | Low: 14, 15, 16  Moderate: 12, 13  High: - |
| Anibarro et al. 2010  Spain | Retrospective review of medical records | Case contacts  (n=599) | - | - | 2HRZ+2HR/4R/6H/9H, SAT  CR: 484/599 = 81% | Low: 12, 13, 14, 15  Moderate: 16 ^D^  High: - |
| Balkhy et al. 2014  Saudi Arabia | Retrospective review of medical records | HCW  (n=50) | - | 9H, SAT  IR: 46/50 = 92%  CR: 8/46 = 17% | - | Low: 12, 14, 15  Moderate: 13, 16^A^  High: - |
| Bandyopadhyay et al. 2002 USA | Retrospective review of medical records | Inmates  (n=150) | - | 6H/12H, SAT  CR: 35/150 = 23% | - | Low: 14, 15,16  Moderate: 12  High: 13 |
| Brassard et al. 2006 Canada | Retrospective review of medical records | Immigrants  (n=484) | - | - | ≤6, 9 or 12H/R, SAT  IR: 377/484 = 78% | Low: 14, 15  Moderate: 13, 16^E^  High: - |
| Cain et al. 2012  USA | Retrospective review of medical records | General population  (n=9090) | - | - | 4R/6H/9H, SAT  IR: 4780/9090 = 53%  CR: 1953/3600 = 54% | Low: 14, 15, 16  Moderate: 13  High: 12 |
|  |  | Immigrants  (n=5759) | - | - | 4R/6H/9H, SAT  IR: 3269/5759 = 57%  CR: 1416/2484 = 57% |  |
| Chang et al. 2014  USA | Retrospective review of medical records | General population  (n=1587) | - | 9H, SAT  CR: 1235/1587 = 78% | - | Low: 12, 14, 16  Moderate: 13, 15  High: - |
| Chee et al. 2004  Singapore | Retrospective review of medical records | Case contacts  (n=1017 ) | - | 6H/9H, SAT  IR: 895/1017 = 88%  CR: 721/895 = 81% | - | Low: 14, 15, 16  Moderate: 13  High: 12 |
| Clerk et al. 2011  UK | Retrospective review of medical records | General population  (n= 57) | 3HR, SAT  CR: 29/43 = 67% | 6H, SAT  CR: 11/14 = 79% | - | Low: 12, 14, 15  Moderate: 13, 16  High: - |
|  |  | Immigrants  (n=38) | - | - | 3HR/6H, SAT  CR: 25/38 = 66% |  |
| Codecasa et al. 2013  Italy | Retrospective review of medical records | General population  (n=11963) | - | 6H, SAT  CR: 8866/11963 = 74% | - | Low: 14, 15, 16  Moderate: 13  High: 12 |
|  |  | Case contacts  (n=6110) | - | 6H, SAT  CR: 4792/6110 = 78% | - |  |
|  |  | HCW  (n=1156) | - | 6H, SAT  CR: 867/1156 = 75% | - |  |
|  |  | Immigrants  (n=8586) | - | 6H, SAT  CR: 6123/8586 = 71% | - |  |
| Cruz et al. 2012  USA | Retrospective review of medical records | General population  (n=289) | - | 6R/9H, SAT/enhanced SAT/DOT  IR: 248/289 = 86%  CR: 186/248 = 75% | - | *For general population:*  Low: 14, 15, 16  Moderate: 12, 13  *For immigrants:*  Low: 14, 15  Moderate: 13, 16  High: 12 |
|  |  | Immigrants  (n=105) | - | 6R/9H, SAT/enhanced SAT/DOT  CR: 71/105 = 68% | - |  |
| Cruz et al. 2013  USA | Retrospective review of medical records | General population  (n=448) | - | 6R/9H, DOT  CR: 429/448 = 96% | - | Low: 12, 13, 14, 15, 16  Moderate: -  High: - |
| Duarte et al. 2012 Portugal | Retrospective review of medical records | General population  (n=514) | 2HRZ, SAT  CR: 301/324 = 93% | 6H, SAT  CR: 171/190 = 90% | - | Low: 14, 15, 16  Moderate: 13  High: 12 |
| Fresard et al. 2011 Switzerland | Retrospective review of medical records | General population  (n= 624) | 4R, SAT  CR: 164/198 = 83% | 6H, SAT  CR: 316/426 = 74% | - | Low: 12, 14, 15  Moderate: 13, 16^F^  High: - |
| Gershon et al. 2004 Canada | Retrospective review of medical records | General population  (n=308) | - | - | According to Canadian Tuberculosis Standards, 5th edition  IR: 179/308 = 58% | Low: 14, 15, 16  Moderate: 16 (for HCW)  High: 13 |
|  |  | HCW  (n=107) | - | - | According to Canadian Tuberculosis Standards, 5th edition  IR: 50/107 = 47% |  |
|  |  | Immigrants  (n=243) | - | - | According to Canadian Tuberculosis Standards, 5th edition  IR: 146/243 = 60% |  |
| Gilroy et al. 2000  USA | Retrospective review of medical records | General population  (n=510) | - | 6H, SAT  IR: 500/510 = 98%  CR: 247/500 = 51% | - | Low: 12, 14, 15, 16  Moderate: 13  High: - |
| Golub et al. 2008  USA | Retrospective review of medical records | PWID  (n=536) | - | 6H, SAT/DOT/SAT +DOT  IR: 299/536 = 56%  CR: 165/299 = 55% | - | Low: 14, 15, 16  Moderate: 13  High: 12 |
| Grinsdale et al. 2011  USA | Retrospective review of medical records | Case contacts  (n=261) | - | - | 4HR/6H/9H, SAT  IR: 205/261 = 79%  CR: 156/205 = 76% | Low: 14, 15, 16  Moderate: 13  High: 12 |
| Haley et al. 2008  USA | Retrospective review of medical records | General population  (n=749) | 4R, SAT  CR: 571/749 = 76% | - | - | Low: 12, 14, 15, 16  Moderate: 13  High: 12 (for groups other than the general population) |
|  |  | Case contacts  (n=93) | 4R, SAT  CR: 59/93 = 63% | - | - |  |
|  |  | Immigrants  (598) | 4R, SAT  CR: 476/598 = 80% | - | - |  |
| Hirsch-Moverman et al. 2010  USA | Retrospective review of medical records | General population  (n=312) | - | 6H/9H/12H, SAT  CR: 139/312 = 45% | - | Low: 14, 15  Moderate: 13, 16^G^  High: 12 |
| Horsburgh et al. 2010 Canada,  USA | Retrospective review of medical records | General population  (IR n=720; CR n=1959) | 2RZ/ 4R, SAT  CR: 67/104 = 64% | 6H/9H, SAT  CR: 856/1855 = 47% | 2RZ/ 4R/6H/9H, SAT  IR: 597/720 = 83% | Low: 14, 15  Moderate: 13, 16  High: 12 |
|  |  | Case contacts  (IR n=212; CR n=401) | - | - | 2RZ/ 4R/6H/9H, SAT  IR: 202/212 = 95%  CR: 193/401 = 48% |  |
|  |  | HCW  (IR n=53; CR n=209) | - | - | 2RZ/ 4R/6H/9H, SAT  IR: 31/53 = 58%  CR: 84/209 = 40% |  |
| Kan et al. 2013  Sweden | Retrospective review of medical records | General population  (n=360) | - | 9H, SAT  CR: 272/360 = 76% | - | Low: 14, 15, 16  Moderate: 13  High: 12 |
|  |  | Case contacts  (n=204) | - | 9H, SAT  CR: 152/204 = 75% | - |  |
|  |  | Immigrants  (n=92) | - | 9H, SAT  CR: 60/92 = 65% | - |  |
| Kwara et al. 2008  USA | Retrospective review of medical records | General population  (n=845) | - | 9H, SAT  IR: 690/845 = 82%  CR: 426/690 = 62% | - | Low: 14, 15, 16  Moderate: 12, 13, 16 (for immigrants)  High: - |
|  |  | Immigrants  (n=99) | - | 9H, SAT  CR: 63/99 = 64% | - |  |
| Langenskiold et al. 2008  Switzerland | Retrospective review of medical records | Case contacts  (n=571) | - | 6H, SAT  IR: 462/571 = 81% | - | Low: 13, 14, 15, 16  Moderate: -  High: - |
| Lardizabal et al. 2006  USA | Retrospective review of medical records | General population  (n=474) | 4R, SAT  CR: 210/261 = 81% | 9H, SAT  CR: 113/213 = 53% | - | Low: 12, 14, 15, 16  Moderate: 13  High: 12 (for groups other than the general population) |
|  |  | Immigrants  (n=432) | - | - | 4R/9H, SAT  CR: 298/432 = 69% |  |
| Lee et al. 2002  USA | Retrospective review of medical records | General population  (n=148) | 2RZ, SAT  CR: 85/148 = 57% | - | - | Low: 12, 14, 15, 16  Moderate: -  High: 13 |
| Li et al. 2010  USA | Retrospective review of medical records | General population  (n=15035) | 4R+RFB, SAT  CR: 603/1005 = 60% | 6H/9H, SAT  CR: 6187/14030 = 44% | - | Low: 12, 14, 15, 16  Moderate: 13, 16 (for HIV-infected)  High: 12 (for groups other than the general population) |
|  |  | Case contacts  (n=2732) | 4R+RFB, SAT  CR: 263/388 = 68% | 6H/9H, SAT  CR: 1306/2344 = 56% | - |  |
|  |  | HIV-infected individuals  (n=95) | - | 6H/9H, SAT  CR: 52/94 = 55% | - |  |
|  |  | Immigrants  (n=12683) | 4R+RFB, SAT  CR: 521/862 = 60% | 6H/9H, SAT  CR: 5237/11821 = 44% | - |  |
| Lincoln et al. 2004  USA | Retrospective review of medical records | Inmates  (n=2127 ) | - | - | 2RZ/6H, SAT  IR: 146/2127 = 7% | Low: 14, 15, 16  Moderate: 13  High: - |
| Lobato et al. 2005  USA | Retrospective review of medical records | Homeless  (n=367) | 2RZ, DOT/ SAT+DOT  CR: 160/367 = 44% | - | - | Low: 12, 13, 14, 15, 16  Moderate: -  High: - |
|  |  | Inmates  (n=844) | 2RZ, DOT/combination SAT DOT  CR: 401/844 = 48% | - | - |  |
| LoBue et al. 2003  USA | Retrospective review of medical records | General population  (n=3788) | - | 6H/9H, SAT  CR: 2414/3788 = 64% | - | Low: 12, 14, 15, 16  Moderate: 13, 16 (for groups other than the general population)  High: 12 (for groups other than the general population and immigrants) |
|  |  | Homeless individuals  (n=48) | - | 6H/9H, SAT  CR: 11/48 = 23% | - |  |
|  |  | Immigrants  (n=2583) | - | 6H/9H, SAT  CR: 1679/2583 = 65% | - |  |
| Lopez et al. 2011  Spain | Retrospective review of medical records | Inmates  (n=902) | 2RZ/3HR/4R, DOT  CR (2RZ): 232/316 = 73%  CR(3HR): 70/82 = 85%  CR (4R): 12/12 = 100% | 9H, DOT  CR: 271/400 = 68% | 2RZ/3HR/4R/9H, DOT  IR: 810/902 = 90% | Low: 12, 13, 14, 15  Moderate: 16^H^  High: - |
| Marks et al. 2000  USA | Retrospective review of medical records | Case contacts  (n=1725) | - | 6H/12H/R, SAT  IR: 129/247 = 52% | - | Low: 14  Moderate: 13, 15, 16  High: - |
| McElroy et al. 2005  USA | Retrospective survey | General population  (n=8087) | 2RZ, SAT  CR: 5145/8087 = 64% | - | - | Low: 15  Moderate: 12, 13, 14, 16^B^  High: - |
| Mugisha et al. 2006 Uganda | Retrospective review of medical records | HIV-infected individuals  (n=894) | - | 9H, SAT  IR: 599/894 = 67%  CR: 335/506 = 66% | - | Low: 12, 14, 15, 16  Moderate: 13  High: - |
| Nuzzo et al. 2013  USA | Retrospective review of medical records | General population  (n=841) | 4R, SAT  CR: 302/379 = 80% | 9H, SAT  CR: 193/267 = 72% | 4R/9H, SAT  IR: 652/841 = 78% | Low: 14, 15, 16  Moderate: 13  High: 12 |
|  |  | Immigrants  (n=595) | 4R, SAT  CR: 273/321 = 85% | 9H, SAT  CR: 136/159 = 86% | 4R/9H, SAT  IR: 485/595 = 82% |  |
| Page et al. 2006  USA | Retrospective review of medical records | General population  (n=2149) | 4R, SAT  CR: 987/1379 = 72% | 9H, SAT (minority DOT)  CR: 405/770 = 53% | - | Low: 14, 15, 16  Moderate: 12, 13  High: - |
|  |  | HCW  (n=136) | - | - | 4R/9H, SAT  CR: 87/136 = 64% |  |
|  |  | Immigrants  (n=1027) | - | - | 4R/9H, SAT  CR: 693/1027 = 67% |  |
| Parsyan et al. 2007  USA | Retrospective review of medical records | General population  (n=1723) | - | 6H/9H, SAT  IR: 1572/1723 = 91%  CR: 607/1572 = 39% | - | Low: 14, 15, 16  Moderate: 13  High: 12 |
|  |  | Immigrants  (n=1222) | - | 6H/9H, SAT  CR: 464/1222 = 38% | - |  |
| Priest et al. 2004  USA | Retrospective review of medical records | Immigrants  (n=423) | 2RZ, DOT  CR: 352/423 = 83% | - | - | Low: 12, 13, 14, 15, 16  Moderate: -  High: - |
| Rennie et al. 2007  UK | Retrospective review of medical records | General population  (n=591) | 3HR, SAT  CR: 189/314 = 60% | 6H, SAT  CR: 127/277 = 46% | - | Low: 13, 15, 16  Moderate: 12, 14  High: *12 (for groups other than the general population)* |
|  |  | Case contacts  (n=247) | - | - | 3HR/6H, SAT  CR: 129/247 = 52% |  |
|  |  | Immigrants  (n=289) | - | - | 3HR/6H, SAT  CR: 153/289 = 53% |  |
| Sanchez-Garcia et al. 2013  Spain | Retrospective review of medical records | Patients with comorbidities^c^  (n=105) | - | 9H, SAT  CR: 79/105 = 75% | - | Low: 14  Moderate: 13, 15 ,16^A, I^  High: 12 |
| Shah et al. 2012  USA | Retrospective review of medical records | General population  (n=842) | - | - | 4R/9HR, SAT  IR: 648/842 = 77% | Low: 14, 15, 16  Moderate: 13  High: - |
| Smith et al. 2011  Canada | Retrospective database study | General population  (n=9145) | 4R, SAT  CR: 258/459 = 56% | 6H, SAT  CR: 4699/8686 = 54% | - | Low: 14, 15, 16  Moderate: 13  High: 12 |
| Stucchi et al. 2012  Brazil | Retrospective review of medical records | Patients with comorbidities^d^  (n=33) | - | 6H, SAT  IR: 27/33 = 82% | - | Low: 14, 15  Moderate: 13, 16^A^  High: - |
| Tavitian et al. 2003  USA | Retrospective review of medical records | HCW  (n=183) | - | - | According to ATS guidelines  IR: 163/183 = 89%  CR: 93/113 = 79% | Low: 12, 14, 15, 16  Moderate: 13  High: - |
| Vinnard et al. 2013  USA | Retrospective review of medical records | General population  (n=219) | - | 9H, SAT  CR: 100/219 = 46% | - | Low: 14, 15  Moderate: 12, 13, 16^J^  High: - |
| White et al. 2003  USA | Retrospective review of medical records | General population  (n=1079) | - | 6H, SAT/DOT  CR (SAT): 447/934 = 48%  CR (DOT): 102/145 = 70% | - | Low: 13, 14, 15, 16  Moderate: 12  High: - |
| Xu et al. 2010  Canada | Retrospective review of medical records | HCW  (n=210) | - | - | 4R/6H, SAT  IR: 165/210 = 79% | Low: 14, 15  Moderate: 13, 16^K^  High: - |
| Young et al. 2009  USA | Retrospective review of medical records | General population  (n=777) | 4R, SAT  CR: 125/138 = 91% | 9H, SAT  CR: 415/639 = 65% | - | Low: 14, 16  Moderate: 13, 15  High: 12 |
| Yun et al. 2007  South Korea | Retrospective review of medical records | Patients with comorbidities^e^  (n=41) | 3HR/4R, SAT  IR: 38/41 = 93%  CR: 35/38 = 92% | - | - | Low: 14, 15  Moderate: 13, 16^A^,  High: 12 |

^a^ No separated results for short/long treatment were presented in the paper. However only 1 patient received long term isonizaid treatment. Excluding this patient, the completion rate for RMP 4 months could be calculated; ^b^ Rheumatic patients; ^c^ Adults with hematologic malignancies; ^d^ Patients with end-stage liver disease; ^e^ Individuals with rheumatoid arthritis or ankylosing spondylitis; ^f^different treatment regimens separated by “/”;^g^ Quality aspects of randomised controlled trials: 1: Randomisation; 2: Allocation concealment; 3: Blinding; 4: Similarity treatment and control group; 5: Intention-to-treat analysis; 6: Drop-outs; 7: Treatment adherence assessment; 8: Other bias. Quality aspects of prospective observational studies: 9: Drop-outs (only applicable for studies presenting completion rates); 10: Treatment adherence assessment; 11: Other bias. Quality aspects of retrospective observational studies: 12: Drop-outs (only applicable for studies presenting completion rates); 13: Treatment adherence assessment; 14: Recall; 15: Retrospective selection; 16: Other bias. The aspects “confounders taken into account” and “confidence intervals provided” were only used for review questions 2 and 3 since no risk factor analyses results were included for review question 1.

ATS: American Thoracic Society; CR: completion rate; DOT; directly observed therapy; H:isoniazid; HCW: healthcare worker; HIV: human immunodeficiency virus; HR: isoniazid and rifiampin/rifampicin; HRZ: isoniazid and rifiampin/rifampicin and pyrazinamide; IR: initiation rate; MMWR: Morbidity and Mortality Weekly Report; PWID: people who inject drugs; R: rifampin/rifampicin; RCT: randomized controlled trial; RFB: rifabutin; RZ: rifiampin/rifampicin and pyrazinamide; SAT; self-administered therapy; USA: United States of America; Z:pyrazinamide.
^A^ Small sample size; ^B^ Limited population characteristics; ^C^ Limited demographic data; ^D^ Participation selection, 2 specialised TB units; ^E^ Results section not clear; ^F^ Study conducted in two different periods, analysis not clear of group who switched treatment regimen; ^G^ Two time periods: inconsistency in recording may have occurred; ^H^ Treatment was indicated as primary chemoprophylaxis for a subgroup (n=40, initiated=20), included in analysis; treatment description changed during study period; ^I^ Individuals with LTBI not detected by the diagnostic tests because of immunocompromised status; ^J^ Unknown how many patients with LTBI did not have a HIV or hepatitis C test; ^K^ Refusal not always documented; ^L^ Interpretation of negative tests is complicated by the effect of immunosuppressant precluding effective testing, not clear how switched treatment is analysed; ^M^ Limited power.

**References**

1. Bastos ML, Menzies D, Belo MT, et al. Changes in QuantiFERON(R)-TB Gold In-Tube results during treatment for tuberculous infection. Int J Tuberc Lung Dis **2013**; 17:909-16.

2. Jasmer RM, Saukkonen JJ, Blumberg HM, et al. Short-course rifampin and pyrazinamide compared with isoniazid for latent tuberculosis infection: a multicenter clinical trial. Ann Intern Med **2002**; 137:640-7.

3. Jimenez-Fuentes MA, de Souza-Galvao ML, Mila Auge C, Solsona Peiro J, Altet-Gomez MN. Rifampicin plus isoniazid for the prevention of tuberculosis in an immigrant population. Int J Tuberc Lung Dis **2013**; 17:326-32.

4. Matteelli A, Olliaro P, Signorini L, et al. Tolerability of twice-weekly rifabutin-isoniazid combinations versus daily isoniazid for latent tuberculosis in HIV-infected subjects: a pilot study. Int J Tuberc Lung Dis **1999**; 3:1043-6.

5. Matteelli A, Casalini C, Raviglione MC, et al. Supervised preventive therapy for latent tuberculosis infection in illegal immigrants in Italy. Am J Respir Crit Care Med **2000**; 162:1653-5.

6. Menzies D, Dion MJ, Rabinovitch B, Mannix S, Brassard P, Schwartzman K. Treatment completion and costs of a randomized trial of rifampin for 4 months versus isoniazid for 9 months. Am J Respir Crit Care Med **2004**; 170:445-9.

7. Menzies D, Long R, Trajman A, et al. Adverse events with 4 months of rifampin therapy or 9 months of isoniazid therapy for latent tuberculosis infection: a randomized trial. Ann Intern Med **2008**; 149:689-97.

8. Rivero A, Lopez-Cortes L, Castillo R, et al. [Randomized clinical trial investigating three chemoprophylaxis regimens for latent tuberculosis infection in HIV-infected patients]. Enferm Infecc Microbiol Clin **2007**; 25:305-10.

9. Schechter M, Zajdenverg R, Falco G, et al. Weekly rifapentine/isoniazid or daily rifampin/pyrazinamide for latent tuberculosis in household contacts. Am J Respir Crit Care Med **2006**; 173:922-6.

10. Spyridis NP, Spyridis PG, Gelesme A, et al. The effectiveness of a 9-month regimen of isoniazid alone versus 3- and 4-month regimens of isoniazid plus rifampin for treatment of latent tuberculosis infection in children: results of an 11-year randomized study. Clin Infect Dis **2007**; 45:715-22.

11. Sterling TR, Villarino ME, Borisov AS, et al. Three months of rifapentine and isoniazid for latent tuberculosis infection. N Engl J Med **2011**; 365:2155-66.

12. Tortajada C, Martinez-Lacasa J, Sanchez F, et al. Is the combination of pyrazinamide plus rifampicin safe for treating latent tuberculosis infection in persons not infected by the human immunodeficiency virus? Int J Tuberc Lung Dis **2005**; 9:276-81.

13. Trajman A, Long R, Zylberberg D, Dion MJ, Al-Otaibi B, Menzies D. Factors associated with treatment adherence in a randomised trial of latent tuberculosis infection treatment. Int J Tuberc Lung Dis **2010**; 14:551-9.

14. Bark CM, Morrison CS, Salata RA, et al. Acceptability of treatment of latent tuberculosis infection in newly HIV-infected young women in Uganda. Int J Tuberc Lung Dis **2010**; 14:1647-9.

15. Bock NN, Metzger BS, Tapia JR, Blumberg HM. A tuberculin screening and isoniazid preventive therapy program in an inner-city population. Am J Respir Crit Care Med **1999**; 159:295-300.

16. Bock NN, Rogers T, Tapia JR, Herron GD, DeVoe B, Geiter LJ. Acceptability of short-course rifampin and pyrazinamide treatment of latent tuberculosis infection among jail inmates. Chest **2001**; 119:833-7.

17. Brassard P, Bruneau J, Schwartzman K, Senecal M, Menzies D. Yield of tuberculin screening among injection drug users. Int J Tuberc Lung Dis **2004**; 8:988-93.

18. Golub JE, Pronyk P, Mohapi L, et al. Isoniazid preventive therapy, HAART and tuberculosis risk in HIV-infected adults in South Africa: a prospective cohort. AIDS **2009**; 23:631-6.

19. Goswami ND, Gadkowski LB, Piedrahita C, et al. Predictors of latent tuberculosis treatment initiation and completion at a U.S. public health clinic: a prospective cohort study. BMC Public Health **2012**; 12:468.

20. Hiransuthikul N, Nelson KE, Hiransuthikul P, Vorayingyong A, Paewplot R. INH preventive therapy among adult HIV-infected patients in Thailand. Int J Tuberc Lung Dis **2005**; 9:270-5.

21. Lashley M. A targeted testing program for tuberculosis control and prevention among Baltimore city's homeless population. Public Health Nurs **2007**; 24:34-9.

22. Lee SH, Yim JJ, Kim HJ, et al. Adverse events and development of tuberculosis after 4 months of rifampicin prophylaxis in a tuberculosis outbreak. Epidemiol Infect **2012**; 140:1028-35.

23. Lorvick J, Thompson S, Edlin BR, Kral AH, Lifson AR, Watters JK. Incentives and accessibility: a pilot study to promote adherence to TB prophylaxis in a high-risk community. J Urban Health **1999**; 76:461-7.

24. Machado A, Jr., Finkmoore B, Emodi K, et al. Risk factors for failure to complete a course of latent tuberculosis infection treatment in Salvador, Brazil. Int J Tuberc Lung Dis **2009**; 13:719-25.

25. Minodier P, Lamarre V, Carle ME, Blais D, Ovetchkine P, Tapiero B. Evaluation of a school-based program for diagnosis and treatment of latent tuberculosis infection in immigrant children. J Infect Public Health **2010**; 3:67-75.

26. Transmission of Mycobacterium tuberculosis associated with failed completion of treatment for latent tuberculosis infection--Chickasaw County, Mississippi, June 1999-March 2002. MMWR Morb Mortal Wkly Rep **2003**; 52:222-4.

27. Morano JP, Walton MR, Zelenev A, Bruce RD, Altice FL. Latent tuberculosis infection: screening and treatment in an urban setting. J Community Health **2013**; 38:941-50.

28. Morisky DE, Ebin VJ, Malotte CK, Coly A, Kominski G. Assessment of tuberculosis treatment completion in an ethnically diverse population using two data sources. Implications for treatment interventions. Eval Health Prof **2003**; 26:43-58.

29. Narita M, Kellman M, Franchini DL, McMillan ME, Hollender ES, Ashkin D. Short-course rifamycin and pyrazinamide treatment for latent tuberculosis infection in patients with HIV infection: the 2-year experience of a comprehensive community-based program in Broward County, Florida. Chest **2002**; 122:1292-8.

30. Nolan CM, Roll L, Goldberg SV, Elarth AM. Directly observed isoniazid preventive therapy for released jail inmates. Am J Respir Crit Care Med **1997**; 155:583-6.

31. Oni T, Tsekela R, Kwaza B, et al. A recent HIV diagnosis is associated with non-completion of Isoniazid Preventive Therapy in an HIV-infected cohort in Cape Town. PLoS One **2012**; 7:e52489.

32. Pettit AC, Bethel J, Hirsch-Moverman Y, Colson PW, Sterling TR. Female sex and discontinuation of isoniazid due to adverse effects during the treatment of latent tuberculosis. J Infect **2013**; 67:424-32.

33. Rutherford ME, Ruslami R, Anselmo M, et al. Management of children exposed to mycobacterium tuberculosis: A public health evaluation in west java, indonesia. Bulletin of the World Health Organization **2013**; 91:932-41.

34. Sadaphal P, Astemborski J, Graham NM, et al. Isoniazid preventive therapy, hepatitis C virus infection, and hepatotoxicity among injection drug users infected with Mycobacterium tuberculosis. Clin Infect Dis **2001**; 33:1687-91.

35. Sarivalasis A, Bodenmann P, Langenskiold E, Lutchmaya-Flick C, Daher O, Zellweger JP. High rate of completion of preventive therapy for latent tuberculosis infection among asylum seekers in a Swiss Canton. Swiss Med Wkly **2013**; 143:w13860.

36. Scholten JN, Driver CR, Munsiff SS, et al. Effectiveness of isoniazid treatment for latent tuberculosis infection among human immunodeficiency virus (HIV)-infected and HIV-uninfected injection drug users in methadone programs. Clin Infect Dis **2003**; 37:1686-92.

37. Shukla SJ, Warren DK, Woeltje KF, Gruber CA, Fraser VJ. Factors associated with the treatment of latent tuberculosis infection among health-care workers at a midwestern teaching hospital. Chest **2002**; 122:1609-14.

38. Snyder DC, Paz EA, Mohle-Boetani JC, Fallstad R, Black RL, Chin DP. Tuberculosis prevention in methadone maintenance clinics. Effectiveness and cost-effectiveness. Am J Respir Crit Care Med **1999**; 160:178-85.

39. Stout JE, Engemann JJ, Cheng AC, Fortenberry ER, Hamilton CD. Safety of 2 months of rifampin and pyrazinamide for treatment of latent tuberculosis. Am J Respir Crit Care Med **2003**; 167:824-7.

40. Trauer JM, Krause VL. Assessment and management of latent tuberculosis infection in a refugee population in the Northern Territory. Med J Aust **2011**; 194:579-82.

41. Valls V, Ena J. Short-course treatment of latent tuberculosis infection in patients with rheumatic conditions proposed for anti-TNF therapy. Clin Rheumatol **2014**.

42. White MC, Tulsky JP, Menendez E, Goldenson J, Kawamura LM. Incidence of TB in inmates with latent TB infection: 5-year follow-up. Am J Prev Med **2005**; 29:295-301.

43. White MC, Tulsky JP, Goldenson J, Portillo CJ, Kawamura M, Menendez E. Randomized controlled trial of interventions to improve follow-up for latent tuberculosis infection after release from jail. Arch Intern Med **2002**; 162:1044-50.

44. Young J, Edick T, Klee D, O'Connor ME. Successful treatment of pediatric latent tuberculosis infection in a community health center clinic. Pediatr Infect Dis J **2012**; 31:e147-51.

45. Ailinger RL, Dear MR. Adherence to tuberculosis preventive therapy among Latino immigrants. Public Health Nurs **1998**; 15:19-24.

46. Ailinger RL, Moore JB, Nguyen N, Lasus H. Adherence to latent tuberculosis infection therapy among latino immigrants. Public Health Nurs **2006**; 23:307-13.

47. Ailinger RL, Black P, Nguyen N, Lasus H. Predictors of Adherence to Latent Tuberculosis Infection Therapy in Latino Immigrants. Journal of Community Health Nursing **2007**; 24:191-8.

48. Anger HA, Proops D, Harris TG, et al. Active case finding and prevention of tuberculosis among a cohort of contacts exposed to infectious tuberculosis cases in New York City. Clin Infect Dis **2012**; 54:1287-95.

49. Anibarro L, Casas S, Paz-Esquete J, et al. Treatment completion in latent tuberculosis infection at specialist tuberculosis units in Spain. Int J Tuberc Lung Dis **2010**; 14:701-7.

50. Balkhy HH, Miller TL, Ali S, et al. Compliance with postexposure screening and treatment of latent tuberculosis infection among healthcare workers in a tertiary care hospital in Saudi Arabia. Infect Control Hosp Epidemiol **2014**; 35:176-81.

51. Bandyopadhyay T, Murray H, Metersky ML. Cost-effectiveness of tuberculosis prophylaxis after release from short-term correctional facilities. Chest **2002**; 121:1771-5.

52. Brassard P, Steensma C, Cadieux L, Lands LC. Evaluation of a school-based tuberculosis-screening program and associate investigation targeting recently immigrated children in a low-burden country. Pediatrics **2006**; 117:e148-56.

53. Cain KP, Garman KN, Laserson KF, et al. Moving toward tuberculosis elimination: implementation of statewide targeted tuberculin testing in Tennessee. Am J Respir Crit Care Med **2012**; 186:273-9.

54. Chang SH, Eitzman SR, Nahid P, Finelli ML. Factors associated with failure to complete isoniazid therapy for latent tuberculosis infection in children and adolescents. J Infect Public Health **2014**; 7:145-52.

55. Chee CB, Teleman MD, Boudville IC, Do SE, Wang YT. Treatment of latent TB infection for close contacts as a complementary TB control strategy in Singapore. Int J Tuberc Lung Dis **2004**; 8:226-31.

56. Clerk N, Sisson K, Antunes G. Latent tuberculosis: concordance and duration of treatment regimens. Br J Nurs **2011**; 20:824-7.

57. Codecasa LR, Murgia N, Ferrarese M, et al. Isoniazid preventive treatment: predictors of adverse events and treatment completion. Int J Tuberc Lung Dis **2013**; 17:903-8.

58. Cruz AT, Starke JR. Increasing adherence for latent tuberculosis infection therapy with health department-administered therapy. Pediatr Infect Dis J **2012**; 31:193-5.

59. Cruz AT, Starke JR. Twice-weekly therapy for children with tuberculosis infection or exposure. Int J Tuberc Lung Dis **2013**; 17:169-74.

60. Duarte R, Carvalho A, Correia A. Two-month regimen of isoniazid, rifampin and pirazinamid for latent tuberculosis infection. Public Health **2012**; 126:760-2.

61. Fresard I, Bridevaux PO, Rochat T, Janssens JP. Adverse effects and adherence to treatment of rifampicin 4 months vs isoniazid 6 months for latent tuberculosis: a retrospective analysis. Swiss Med Wkly **2011**; 141:w13240.

62. Gershon AS, McGeer A, Bayoumi AM, Raboud J, Yang J. Health care workers and the initiation of treatment for latent tuberculosis infection. Clin Infect Dis **2004**; 39:667-72.

63. Gilroy SA, Rogers MA, Blair DC. Treatment of latent tuberculosis infection in patients aged > or =35 years. Clin Infect Dis **2000**; 31:826-9.

64. Golub JE, Astemborski J, Ahmed M, et al. Long-term effectiveness of diagnosing and treating latent tuberculosis infection in a cohort of HIV-infected and at-risk injection drug users. J Acquir Immune Defic Syndr **2008**; 49:532-7.

65. Grinsdale JA, Ho CS, Banouvong H, Kawamura LM. Programmatic impact of using QuantiFERON(R)-TB Gold in routine contact investigation activities. Int J Tuberc Lung Dis **2011**; 15:1614-20.

66. Haley CA, Stephan S, Vossel LF, Sherfy EA, Laserson KF, Kainer MA. Successful use of rifampicin for Hispanic foreign-born patients with latent tuberculosis infection. Int J Tuberc Lung Dis **2008**; 12:160-7.

67. Hirsch-Moverman Y, Bethel J, Colson PW, Franks J, El-Sadr W. Predictors of latent tuberculosis infection treatment completion in the United States: an inner city experience. Int J Tuberc Lung Dis **2010**; 14:1104-11.

68. Horsburgh CR, Jr., Goldberg S, Bethel J, et al. Latent TB infection treatment acceptance and completion in the United States and Canada. Chest **2010**; 137:401-9.

69. Kan B, Kalin M, Bruchfeld J. Completing treatment for latent tuberculosis: patient background matters. Int J Tuberc Lung Dis **2013**; 17:597-602.

70. Kwara A, Herold JS, Machan JT, Carter EJ. Factors associated with failure to complete isoniazid treatment for latent tuberculosis infection in Rhode Island. Chest **2008**; 133:862-8.

71. Langenskiold E, Herrmann FR, Luong BL, Rochat T, Janssens JP. Contact tracing for tuberculosis and treatment for latent infection in a low incidence country. Swiss Med Wkly **2008**; 138:78-84.

72. Lardizabal A, Passannante M, Kojakali F, Hayden C, Reichman LB. Enhancement of treatment completion for latent tuberculosis infection with 4 months of rifampin. Chest **2006**; 130:1712-7.

73. Lee AM, Mennone JZ, Jones RC, Paul WS. Risk factors for hepatotoxicity associated with rifampin and pyrazinamide for the treatment of latent tuberculosis infection: experience from three public health tuberculosis clinics. Int J Tuberc Lung Dis **2002**; 6:995-1000.

74. Li J, Munsiff SS, Tarantino T, Dorsinville M. Adherence to treatment of latent tuberculosis infection in a clinical population in New York City. Int J Infect Dis **2010**; 14:e292-7.

75. Lincoln T, Brannan GL, Lynch V, et al. Completing tuberculosis prophylaxis in jail: targeting treatment and comparison of rifampin/pyrazinamide with isoniazid regimens. Int J Tuberc Lung Dis **2004**; 8:306-11.

76. Lobato MN, Reves RR, Jasmer RM, Grabau JC, Bock NN, Shang N. Adverse events and treatment completion for latent tuberculosis in jail inmates and homeless persons. Chest **2005**; 127:1296-303.

77. LoBue PA, Moser KS. Use of isoniazid for latent tuberculosis infection in a public health clinic. Am J Respir Crit Care Med **2003**; 168:443-7.

78. Lopez G, Wood M, Ayesta FJ. [10 years of innovation in the treatment of latent tuberculosis infection: a comparison between standard and short course therapies in directly observed therapy]. Rev Esp Sanid Penit **2011**; 13:3-14.

79. Marks SM, Taylor Z, Qualls NL, Shrestha-Kuwahara RJ, Wilce MA, Nguyen CH. Outcomes of contact investigations of infectious tuberculosis patients. Am J Respir Crit Care Med **2000**; 162:2033-8.

80. McElroy PD, Ijaz K, Lambert LA, et al. National survey to measure rates of liver injury, hospitalization, and death associated with rifampin and pyrazinamide for latent tuberculosis infection. Clin Infect Dis **2005**; 41:1125-33.

81. Mugisha B, Bock N, Mermin J, et al. Tuberculosis case finding and preventive therapy in an HIV voluntary counseling and testing center in Uganda. Int J Tuberc Lung Dis **2006**; 10:761-7.

82. Nuzzo JB, Golub JE, Chaulk P, Shah M. Analysis of Latent Tuberculosis Infection Treatment Adherence Among Refugees and Other Patient Groups Referred to the Baltimore City Health Department TB Clinic, February 2009-March 2011. J Immigr Minor Health **2013**.

83. Page KR, Sifakis F, Montes de Oca R, et al. Improved adherence and less toxicity with rifampin vs isoniazid for treatment of latent tuberculosis: a retrospective study. Arch Intern Med **2006**; 166:1863-70.

84. Parsyan AE, Saukkonen J, Barry MA, Sharnprapai S, Horsburgh CR, Jr. Predictors of failure to complete treatment for latent tuberculosis infection. J Infect **2007**; 54:262-6.

85. Priest DH, Vossel LF, Jr., Sherfy EA, Hoy DP, Haley CA. Use of intermittent rifampin and pyrazinamide therapy for latent tuberculosis infection in a targeted tuberculin testing program. Clin Infect Dis **2004**; 39:1764-71.

86. Rennie TW, Bothamley GH, Engova D, Bates IP. Patient choice promotes adherence in preventive treatment for latent tuberculosis. Eur Respir J **2007**; 30:728-35.

87. Sanchez-Garcia EM, Gamallo R, Blanco-Moure A, Viejo MA, Amador L, Anibarro L. Toxicity and adherence to treatment for latent tuberculosis infection in patients with hematologic malignancies. Infection **2013**; 41:903-7.

88. Shah M, DiPietro D, Greenbaum A, et al. Programmatic impact of QuantiFERON-TB Gold In-Tube implementation on latent tuberculosis diagnosis and treatment in a public health clinic. PLoS ONE **2012**; 7:e36551.

89. Smith BM, Schwartzman K, Bartlett G, Menzies D. Adverse events associated with treatment of latent tuberculosis in the general population. CMAJ **2011**; 183:E173-9.

90. Stucchi RS, Boin IF, Angerami RN, Zanaga L, Ataide EC, Udo EY. Is isoniazid safe for liver transplant candidates with latent tuberculosis? Transplant Proc **2012**; 44:2406-10.

91. Tavitian SM, Spalek VH, Bailey RP. A pharmacist-managed clinic for treatment of latent tuberculosis infection in health care workers. Am J Health Syst Pharm **2003**; 60:1856-61.

92. Vinnard C, Gopal A, Linkin DR, Maslow J. Isoniazid Toxicity among an Older Veteran Population: A Retrospective Cohort Study. Tuberc Res Treat **2013**; 2013:549473.

93. White MC, Gournis E, Kawamura M, Menendez E, Tulsky JP. Effect of directly observed preventive therapy for latent tuberculosis infection in San Francisco. Int J Tuberc Lung Dis **2003**; 7:30-5.

94. Xu Y, Schwartzman K. Referrals for positive tuberculin tests in new health care workers and students: a retrospective cohort study. BMC Public Health **2010**; 10:28.

95. Young H, Wessolossky M, Ellis J, Kaminski M, Daly JS. A retrospective evaluation of completion rates, total cost, and adverse effects for treatment of latent tuberculosis infection in a public health clinic in central massachusetts. Clin Infect Dis **2009**; 49:424-7.

96. Yun JW, Lim SY, Suh GY, et al. Diagnosis and treatment of latent tuberculosis infection in arthritis patients treated with tumor necrosis factor antagonists in Korea. J Korean Med Sci **2007**; 22:779-83.
