# Supplementary material for: Initiation and completion rates for latent tuberculosis infection treatment: a systematic review
Source: BMC Infect Dis. 2016 May 17;16:204. doi: 10.1186/s12879-016-1550-y (PMC4869320; doi:10.1186/s12879-016-1550-y)
Supplement: Additional file 4: — Forest plots. (DOC 396 kb) [file 12879_2016_1550_MOESM4_ESM.doc]

# Additional file 4: Forest plots

| **Design** | **Study** | **Duration regimen** | 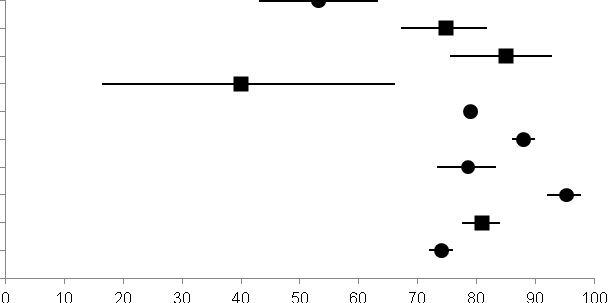**Initiation rates – Case contacts with LTBI** | **% (95%CI)** |
| --- | --- | --- | --- | --- |
| **Prospective** | Goswami et al., 2012 | Combined |  | 53 (43-63) |
|  | Machado et al.,2009 | Long |  | 75 (67-82) |
|  | MMWR. March 21, 2003 | Long |  | 85 (75-93) |
|  | Rutherford et al., 2013 | Long |  | 40 (16-66) |
| **Retrospective** | Anger et al., 2012 | Combined |  | 79 (78-80) |
|  | Chee et al., 2004 | Combined |  | 88 (86-90) |
|  | Grinsdale et al., 2011 | Combined |  | 79 (73-83) |
|  | Horsburgh et al., 2010 | Combined |  | 95 (92-98) |
|  | Langenskiold et al., 2008 | Long |  | 81 (78-84) |
|  | Marks et al., 2000 | Combined |  | 74 (72-76) |

- LTBI: latent tuberculosis infection
  Circle: combined, square: long, triangle: short

| **Design** | **Study** | 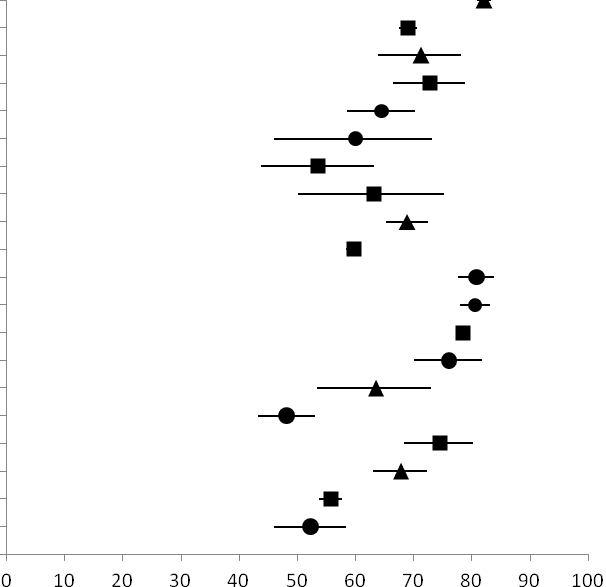**Duration regimen** | **Completion rates – Case contacts with LTBI** | **% (95%CI)** |
| --- | --- | --- | --- | --- |
| **RCT** | Sterling et al., 2011 | Short |  | 82 (81-83) |
|  | Sterling et al., 2011 | Long |  | 69 (68-70) |
|  | Tortajada et al., 2005 | Short |  | 71 (64-78) |
|  | Tortajada et al., 2005 | Long |  | 73 (66-79) |
|  | Trajman et al., 2010 | Combined |  | 64 (59-70) |
| **Prospective** | Goswami et al., 2012 | Combined |  | 60 (46-73) |
|  | Machado et al., 2009 | Long |  | 53 (44-63) |
|  | MMWR. March 21, 2003 | Long |  | 63 (50-75) |
| **Retrospective** | Anger et al., 2012 | Short |  | 69 (65-72) |
|  | Anger et al., 2012 | Long |  | 60 (58-61) |
|  | Anibarro et al., 2010 | Combined |  | 81 (78-84) |
|  | Chee et al., 2004 | Combined |  | 81 (78-83) |
|  | Codecasa et al., 2013 | Long |  | 78 (77-79) |
|  | Grinsdale et al., 2011 | Combined |  | 76 (70-82) |
|  | Haley et al., 2008 | Short |  | 63 (53-73) |
|  | Horsburgh et al., 2010 | Combined |  | 48 (43-53) |
|  | Kan et al., 2013 | Long |  | 75 (68-80) |
|  | Li et al., 2010 | Short |  | 68 (63-72) |
|  | Li et al., 2010 | Long |  | 56 (54-58) |
|  | Rennie et al., 2007 | Combined |  | 52 (46-58) |

- Circle: combined, square: long, triangle: short

| **Design** | **Study** | **Duration regimen** | **Initiation rates – Healthcare workers with LTBI** | **% (95%CI)** |
| --- | --- | --- | --- | --- |
| **Prospective** | Shukla et al., 2002 | 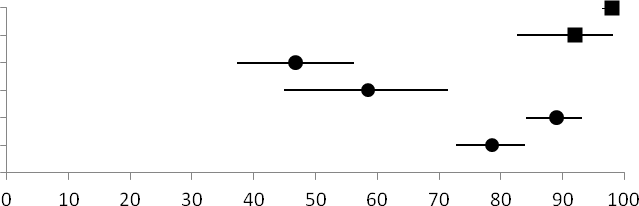Long |  | 98 (96-99) |
| **Retrospective** | Balkhy et al., 2014 | Long |  | 92 (83-98) |
|  | Gershon et al., 2004 | Combined |  | 47 (37-56) |
|  | Horsburgh et al., 2010 | Combined |  | 58 (45-71) |
|  | Tavitian et al., 2003 | Combined |  | 89 (84-93) |
|  | Xu et al., 2010 | Combined |  | 79 (73-84) |

- LTBI: latent tuberculosis infection
  Circle: combined, square: long

| **Design** | **Study** | **Duration regimen** | **Completion rates – Healthcare workers with LTBI** | **% (95%CI)** |
| --- | --- | --- | --- | --- |
| **Prospective** | Pettit et al., 2013 | Combined | 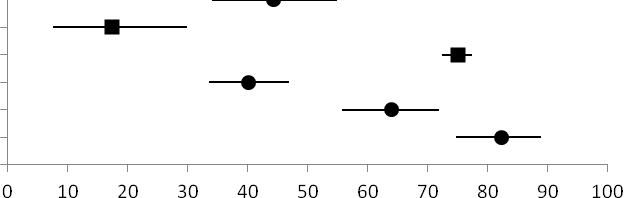 | 44 (34-55) |
| **Retrospective** | Balkhy et al., 2014 | Long |  | 17 (8-30) |
|  | Codecasa et al., 2013 | Long |  | 75 (72-77) |
|  | Horsburgh et al., 2010 | Combined |  | 40 (34-47) |
|  | Page et al., 2006 | Combined |  | 64 (56-72) |
|  | Tavitian et al., 2003 | Combined |  | 82 (75-89) |

- LTBI: latent tuberculosis infection
  Circle: combined, square: long

| **Design** | **Study** | **Duration regimen** | 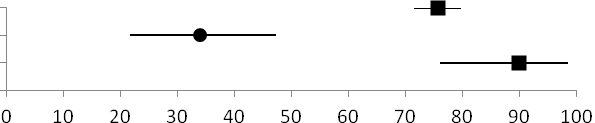**Initiation rates – Homeless with LTBI** | **% (95%CI)** |
| --- | --- | --- | --- | --- |
| **Prospective** | Bock et al., 1999 | Long |  | 76 (72-80) |
|  | Goswami et al., 2012 | Combined |  | 34 (22-47) |
|  | Lashley et al., 2006 | Long |  | 90 (76-99) |

- LTBI: latent tuberculosis infection
  Circle: combined, square: long

| **Design** | **Study** | **Duration regimen** | **Completion rates – Homeless with LTBI** | **% (95%CI)** |
| --- | --- | --- | --- | --- |
| **Prospective** | Goswami et al., 2012 | 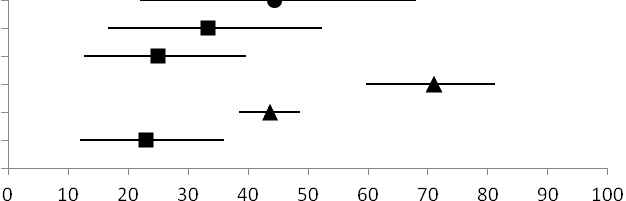Combined |  | 44 (22-68) |
|  | Lashley et al., 2006 | Long |  | 33 (17-52) |
|  | Pettit et al., 2013 | Long |  | 25 (13-40) |
|  | Stout et al., 2003 | Short |  | 71 (60-81) |
| **Retrospective** | Lobato et al., 2005 | Short |  | 44 (39-49) |
|  | LoBue et al., 2003 | Long |  | 23 (12-36) |

- LTBI: latent tuberculosis infection
  Circle: combined, square: long, triangle: short

| **Design** | **Study** | 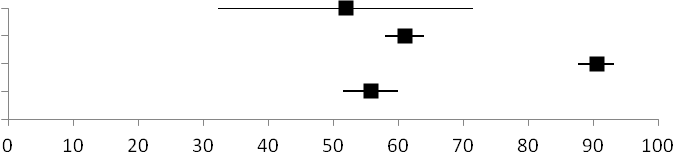**Duration regimen** | **Initiation rates – People who inject drugs with LTBI** | **% (95%CI)** |
| --- | --- | --- | --- | --- |
| **Prospective** | Brassard et al., 2004 | Long |  | 52 (32-71) |
|  | Scholten et al., 2003 | Long |  | 61 (58-64) |
|  | Snyder et al., 1999 | Long |  | 91 (88-93) |
| **Retrospective** | Golub et al., 2008 | Long |  | 56 (52-60) |

- LTBI: latent tuberculosis infection
  Square: long, triangle: short

| **Design** | **Study** | **Duration regimen** | 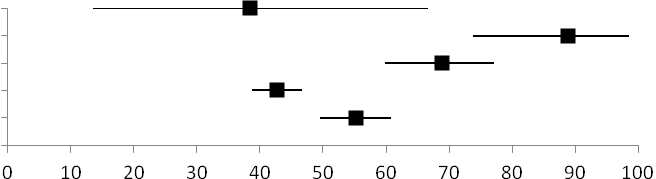**Completion rates – People who inject drugs with LTBI** | **% (95%CI)** |
| --- | --- | --- | --- | --- |
| **Prospective** | Brassard et al., 2004 | Long |  | 38 (14-67) |
|  | Lorvick et al., 1999 | Long |  | 89 (74-98) |
|  | Sadaphal et al., 2001 | Long |  | 69 (60-77) |
|  | Scholten et al., 2003 | Long |  | 43 (39-47) |
| **Retrospective** | Golub et al., 2008 | Long |  | 55 (50-61) |

- LTBI: latent tuberculosis infection
  Square: long

| **Design** | **Study** | **Duration regimen** | 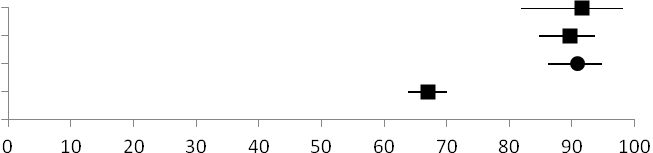**Initiation rates – HIV-positives with LTBI** | **% (95%CI)** |
| --- | --- | --- | --- | --- |
| **Prospective** | Bark et al., 2010 | Long |  | 92 (82-98) |
|  | Oni et al., 2007 | Long |  | 90 (85-94) |
|  | Narita et al., 2002 | Combined |  | 91 (86-95) |
| **Retrospective** | Mugisha et al., 2006 | Long |  | 67 (64-70) |

- LTBI: latent tuberculosis infection
  Circle: combined, square: long

| **Design** | **Study** | 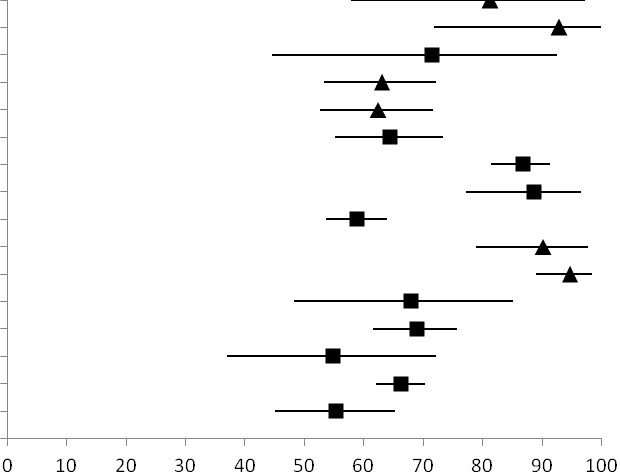**Duration regimen** | **Completion rates – HIV-positives with LTBI** | **% (95%CI)** |
| --- | --- | --- | --- | --- |
| **RCT** | Matteelli et al., 1999 (1) | Short |  | 81 (58-97) |
|  | Matteelli et al., 1999 (2) | Short |  | 93 (72-100) |
|  | Matteelli et al., 1999 | Long |  | 71 (45-93) |
|  | Rivero et al., 2007 (1) | Short |  | 63 (53-72) |
|  | Rivero et al., 2007 (2) | Short |  | 62 (53-72) |
|  | Rivero et al., 2007 | Long |  | 64 (55-73) |
|  | Hiransuthikul et al., 2005 | Long |  | 87 (81-91) |
| **Prospective** | Bark et al., 2010 | Long |  | 89 (77-97) |
|  | Golub et al., 2009 | Long |  | 59 (54-64) |
|  | Narita et al., 2002 (1) | Short |  | 90 (79-98) |
|  | Narita et al., 2002 (2) | Short |  | 95 (89-98) |
|  | Narita et al., 2002 | Long |  | 68 (48-85) |
|  | Oni et al., 2007 | Long |  | 69 (62-76) |
|  | Pettit et al., 2013 | Long |  | 55 (37-72) |
| **Retrospective** | Mugisha et al., 2006 | Long |  | 66 (62-70) |
|  | Li et al., 2010 | Long |  | 55 (45-65) |

- LTBI: latent tuberculosis infection
  Square: long, triangle: short

| **Design** | **Study** | **Duration regimen** | 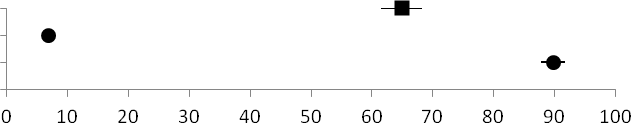**Initiation rates – Inmates with LTBI** | **% (95%CI)** |
| --- | --- | --- | --- | --- |
| **Prospective** | Nolan et al., 1997 | Long |  | 65 (61-68) |
| **Retrospective** | Lincoln et al., 2004 | Combined |  | 7 (6-8) |
|  | Lopez et al., 2011 | Combined |  | 90 (88-92) |

- LTBI: latent tuberculosis infection
  Circle: combined, square: long
-
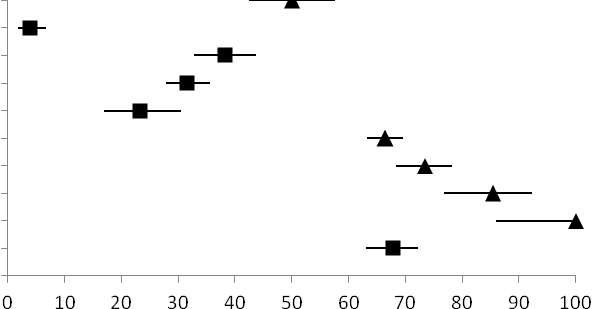


| **Design** | **Study** | **Duration regimen** | **Completion rates – Inmates with LTBI** | **% (95%CI)** |
| --- | --- | --- | --- | --- |
| **Prospective** | Bock et al., 2001 | Short |  | 50 (42-58) |
|  | Bock et al., 2001 | Long |  | 4 (2-7) |
|  | Nolan et al., 1997 | Long |  | 38 (33-44) |
|  | White et al., 2005 | Long |  | 32 (28-36) |
| **Retrospective** | Bandyopadhyay et al., 2002 | Long |  | 23 (17-30) |
|  | Lobato et al., 2005 | Short |  | 66 (63-70) |
|  | Lopez et al., 2011 (1) | Short |  | 73 (68-78) |
|  | Lopez et al., 2011 (2) | Short |  | 85 (77-92) |
|  | Lopez et al., 2011 (3) | Short |  | 100 (86-100) |
|  | Lopez et al., 2011 | Long |  | 68 (63-72) |

- LTBI: latent tuberculosis infection
  Square: long, triangle: short

| **Design** | **Study** | **Duration regimen** | **Initiation rates – Immigrants with LTBI** | **% (95%CI)** |
| --- | --- | --- | --- | --- |
| **Prospective** | Goswami et al., 2012 | 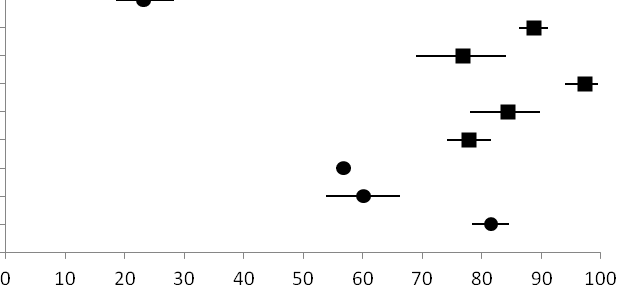Combined |  | 23 (18-28) |
|  | Minodier et al., 2010 | Long |  | 89 (86-91) |
|  | Trauer & Krause et al., 2011 | Long |  | 77 (69-84) |
|  | Young et al., 2012 | Long |  | 97 (94-99) |
| **Retrospective** | Ailinger et al., 2007 | Long |  | 84 (78-90) |
|  | Brassard et al., 2006 | Long |  | 78 (74-81) |
|  | Cain et al., 2012 | Combined |  | 57 (55-58) |
|  | Gershon et al., 2004 | Combined |  | 60 (54-66) |
|  | Nuzzo et al., 2013 | Combined |  | 82 (78-85) |

- LTBI: latent tuberculosis infection
  Circle: combined, square: long

| **Design** | **Study** | 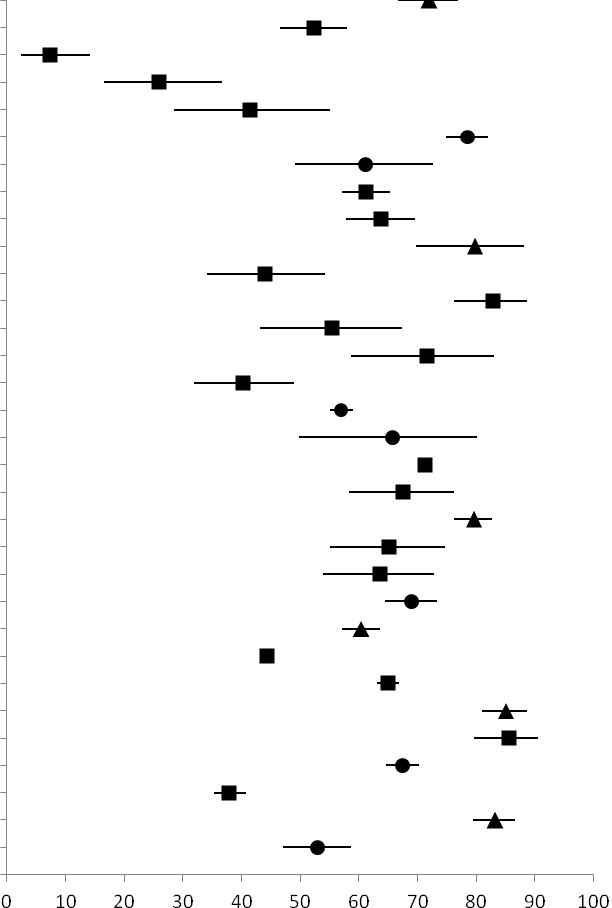**Duration regimen** | **Completion rates – Immigrants with LTBI** | **% (95%CI)** |
| --- | --- | --- | --- | --- |
| **RCT** | Jiménez-Fuentez et al., 2013 | Short |  | 72 (67-77) |
|  | Jiménez-Fuentez et al., 2013 | Long |  | 52 (47-58) |
|  | Matteelli et al., 2000 (1) | Long |  | 7 (3-14) |
|  | Matteelli et al., 2000 (2) | Long |  | 26 (17-37) |
|  | Matteelli et al., 2000 (3) | Long |  | 42 (29-55) |
|  | Trajman et al., 2010 | Combined |  | 79 (75-82) |
| **Prospective** | Goswami et al., 2012 | Combined |  | 61 (49-73) |
|  | Minodier et al., 2010 | Long |  | 61 (57-65) |
|  | Morisky et al., 2003 | Long |  | 64 (58-70) |
|  | Sarivalasis et al., 2013 | Short |  | 80 (70-88) |
|  | Trauer & Krause et al., 2011 | Long |  | 44 (34-54) |
|  | Young et al., 2012 | Long |  | 83 (76-89) |
| **Retrospective** | Ailinger et al., 1998 | Long |  | 55 (43-67) |
|  | Ailinger et al., 2006 | Long |  | 72 (59-83) |
|  | Ailinger et al., 2007 | Long |  | 40 (32-49) |
|  | Cain et al., 2012 | Combined |  | 57 (55-59) |
|  | Clerk et al., 2011 | Combined |  | 66 (50-80) |
|  | Codecasa et al., 2013 | Long |  | 71 (70-72) |
|  | Cruz et al., 2012 | Long |  | 68 (58-76) |
|  | Haley et al., 2008 | Short |  | 80 (76-83) |
|  | Kan et al., 2013 | Long |  | 65 (55-75) |
|  | Kwara et al., 2008 | Long |  | 64 (54-73) |
|  | Lardizabal et al., 2006 | Combined |  | 69 (65-73) |
|  | Li et al., 2010 | Short |  | 60 (57-64) |
|  | Li et al., 2010 | Long |  | 44 (43-45) |
|  | LoBue et al., 2003 | Long |  | 65 (63-67) |
|  | Nuzzo et al., 2013- | Short |  | 85 (81-89) |
|  | Nuzzo et al., 2013 | Long |  | 86 (80-91) |
|  | Page et al., 2006 | Combined |  | 67 (65-70) |
|  | Parsyan et al., 2007 | Long |  | 38 (35-41) |
|  | Priest et al., 2004 | Short |  | 83 (79-87) |
|  | Rennie et al., 2007 | Combined |  | 53 (47-59) |

- LTBI: latent tuberculosis infection
  Circle: combined, square: long, triangle: short
